# Brain connectivity in the left frontotemporal network dynamically modulated by processing difficulty: Evidence from Chinese relative clauses

**DOI:** 10.1371/journal.pone.0230666

**Published:** 2020-04-09

**Authors:** Kunyu Xu, Jeng-Ren Duann

**Affiliations:** 1 Institute of Cognitive Neuroscience, National Central University, Taoyuan, Taiwan; 2 Institute for Neural Computation, University of California San Diego, La Jolla, CA, United States of America; Vanderbilt University Medical Center, UNITED STATES

## Abstract

Although the connection between the left inferior frontal gyrus (LIFG) and the left superior temporal gyrus (LSTG) has been found to be essential for the comprehension of relative clause (RC) sentences, it remains unclear how the LIFG and the LSTG interact with each other, especially during the processing of Chinese RC sentences with different processing difficulty. This study thus conducted a 2 × 2 (modifying position × extraction position) factorial analyses to examine how these two factors influences regional brain activation. The results showed that, regardless of the modifying position, greater activation in the LIFG was consistently elicited in Chinese subject-extracted relative clauses (SRCs) with non-canonical word order than object-extracted relative clauses (ORCs) with canonical word order, implying that the LIFG subserving the ordering process primarily contributes to the processing of information with increased integration demands due to the non-canonical sequence. Moreover, the directional connection between the LIFG and the LSTG appeared to be modulated by different modifying positions. When the RC was at the subject-modifying position, the effective connectivity from the LIFG to the LSTG was dominantly activated for sentence comprehension; whereas when the RC was at the object-modifying position thus being more difficult, it might be the feedback mechanism from the LSTG back to the LIFG that took place in sentence processing. These findings reveal that brain activation in between the LIFG and the LSTG may be dynamically modulated by different processing difficulty and suggest the relative specialization but extensive collaboration involved in the LIFG and the LSTG for sentence comprehension.

## Introduction

One of the challenging questions in the neuroscience of language has been how the human brain processes syntactically complex sentences. Typically, expressing the core meaning of a sentence relies on the linking of the verb and its arguments. During this process, two aspects of processing, storage and ordering, are thought to be crucial for deriving the interpretation of who is doing what to whom [1–3]. First, words in a sentence need to be accessed in sequence and temporarily stored in working memory; second, the accessed words need to be ordered and integrated until relations among the words can be established. Accordingly, a longer argument-verb distance and non-canonical word order substantially induce more storage and ordering costs, resulting in greater processing difficulty for sentence comprehension [3, 4]. Extensive evidence in support of this claim has been collected from the comparison of sentences with subject-extracted relative clauses (SRCs) (e.g., the reporter [*that attacked the senator*] admitted the error) and those with object-extracted relative clauses (ORCs) (e.g., the reporter [*that the senator attacked*] admitted the error). The SRCs and the ORCs share the same lexical content but different word order; thus, there is a well-known processing asymmetry between the SRCs and the ORCs due to the word order effect. For example, English ORCs, which induced larger storage and ordering costs due to non-canonical word order (i.e., object + subject + verb), were consistently found to be more difficult to comprehend than English SRCs with canonical word order [4–7].

Moreover, recent neuroimaging studies have claimed that the storage and ordering processing may be supported by the left superior temporal gyrus (LSTG) and the inferior frontal gyrus (LIFG), respectively [1, 2, 8, 9]. For instance, increased activation in the LIFG is commonly found during the processing of complex sentences that require a hierarchical ordering due to non-canonical word order, compared with the sentences with adjacent elements in canonical order [1, 3, 10, 11]. The role of the LIFG in ordering was even attested to be causal by using repetitive transcranial magnetic stimulation [2]. Lesion studies have also reported that patients with damage in the left frontal region have difficulty in processes of initial syntactic building [12–14]. Thus, it is more likely that the LIFG plays an important role in syntactic parse, such as ordering for the construction of argument hierarchy [3, 8, 10, 15–17]. On the other hand, activation in the LSTG is significantly increased during the processing of sentences with a longer argument-verb distance in comparison to those with a shorter distance, reflecting higher storage costs during sentence processing [1, 2, 4, 6, 10]. Damages to the LSTG may also result in memory decline and sentence processing deficits [18, 19]. It is thus speculated that the LSTG is mainly engaged in short-term storage of argument-verb relations [3, 8, 20–24] or to mediate the integration of semantic and syntactic information [8, 17, 22, 25].

In addition to the respective roles of the LIFG and the LSTG during the processing of sentences, enhanced effective connectivity between the LIFG and the LSTG is also reported to be closely associated with more efficient processing of sentences [26]. The LIFG is even suggested to be the driving input to support word order analysis during complex sentence processing [27]. In our previous study [28], we also found that the processing of more difficult Chinese SRCs rather than the ORCs was supported by the connection from the LIFG to the LSTG. Because in Chinese SRCs (e.g., [*renshi zhangsan de siji*] weifanle guiding), the relative clause (RC) did not follow canonical word order (i.e. verb + object + subject), greater processing costs were required during the storage and ordering processing to construct argument-verb relations. For this reason, increased activation in and between the LIFG and the LSTG was inferred to facilitate the comprehension of the SRCs. On the other hand, for the ORCs with canonical word order (e.g., [*zhangsan renshi de siji*] weifanle guiding), incremental integration of all the upcoming words was sufficient for the reading; hence, less activation in between the LIFG and the LSTG was involved in the processing of the ORCs compared to the SRCs.

Based on accumulating evidence, it appeared that complex sentence processing was probably driven by word-order analysis, supported by the LIFG [27, 28]. However, some concerns may challenge this assumption because increased brain activation in Chinese RC processing may be partially due to the temporal ambiguity between a complement clause analysis and a head-final RC analysis for Chinese SRC construction, rather than the word order effect between Chinese SRCs and ORCs [29, 30]. Therefore, we here investigated another set of Chinese RCs, object-modifying relative clauses (OM-RCs) in which the RCs were used to modify the matrix object of the sentence (as examples shown in Table 1 below) to remove the abovementioned concern. If the processing asymmetry between Chinese SRCs and ORCs were mainly attributed to the word order effect rather than the ambiguity resolution, we would expect to replicate our previous findings by applying the same data analysis (i.e., the conventional fMRI analysis and Granger causality analysis). We hypothesized that greater activation in the LIFG and the LSTG should be found in the processing of the object-modifying subject-extracted relative clauses (OM-SRCs) with non-canonical word order, compared to the object-modifying object-extracted relative clauses (OM-ORCs) with canonical word order. Moreover, through the application of Granger causality analysis, we would examine whether the processing of the RCs is driven by word-order analysis, which is supported by the LIFG. Because both types of the OM-RC sentences are initiated by the same noun-verb sequence (as shown in Table 1A and 1B), followed by a verb-noun-relativizer sequence in an SRC or by a noun-verb-relativizer sequence in an ORC, we hypothesized that the directional connectivity between the LIFG and the LSTG might be altered to account for different processing costs due to incremental nature of sentence processing. Specifically, the processing of the OM-RC sentences might be driven by the LSTG rather than the LIFG for the storage function. Besides, we would conduct a 2 × 2 factorial analysis by using the newly-collected data from the OM-RC sentences, as well as our previously-published data from the subject-modifying relative clause (SM-RC) sentences [28]. The 2 × 2 factorial analysis involved the modifying position (subject- versus object-modifying) and the extraction position (subject-extracted versus object-extracted) to see whether and how these two factors influence regional brain activation.

**Table 1:** The examples of OM-SRC, OM-ORC, SM-SRC, and SM-ORC sentences.

A. Object-modifying subject-extracted relative clause (OM-SRC)					
攝影師	誣陷	認識	張三	的	司機
shèyǐngshī	wūxiàn	rènshi	Zhāngsān	de	sījī
photographer	frame	know	Zhangsan	relativizer	driver
*The photographer framed the driver who knew Zhangsan*.					
B. Object-modifying object-extracted relative clause (OM-ORC)					
攝影師	誣陷	張三	認識	的	司機
shèyǐngshī	wūxiàn	Zhāngsān	Rènshi	de	sījī
photographer	frame	Zhangsan	Know	relativizer	driver
*The photographer framed the driver who Zhangsan knew*.					
C. Subject-modifying subject-extracted relative clause (SM-SRC)					
認識	張三	的	司機	違反了	規定
rènshi	Zhāngsān	de	sījī	wěifǎnle	guīdìng
know	Zhangsan	relativizer	driver	violate	rule
*The driver who knew Zhangsan violated the rules*.					
D. Subject-modifying object-extracted relative clause (SM-ORC)					
張三	認識	的	司機	違反了	規定
Zhāngsān	rènshi	de	sījī	wěifǎnle	guīdìng
Zhangsan	know	relativizer	driver	violate	rule
*The driver who Zhangsan knew violated the rules*.					

Together, in addition to unraveling the neural correlates underlying the processing asymmetry between Chinese SRCs and ORCs, in the present study we also aimed to investigate the information flow between the LIFG and the LSTG and attempted to answer which of the directional connections between the LIFG and the LSTG could be modulated by different processing difficulty involved in different types of RC sentences.

## Materials and methods

### Participants

Nineteen healthy, right-handed native Chinese speakers were paid to participate in this study (10 females; aged from 21 to 32 years, mean age = 24.45 years, standard deviation = 3.10). All participants had normal or corrected-to-normal vision and no history of neurological or psychiatric disorders. The experimental protocol for the study was approved by the Institutional Review Board (IRB) of China Medical University Hospital, Taichung, Taiwan (http://www.cmuh.cmu.edu.tw, Approval No. DMR100-IRB-221). Signed written informed consent was obtained from all the participants before the experiment.

### Materials and design

In this study, there were 64 pairs of Chinese OM-SRC and OM-ORC sentences (see examples in Table 1) in which the matrix object of the sentences was modified by either an SRC (Table 1A) or an ORC (Table 1B). All the sentences were equal in overall word length across different conditions, and each sentence was split into six frames (with each frame containing one to three characters). A pilot study of plausibility rating with another group of 20 participants who did not take part in this fMRI study showed no significant difference in the naturalness of these two types of sentences (*t*_*1*_ (19) = 1.311, p = 0.205; *t*_*2*_ (126) = 0.762, p = 0.448).

These 64 pairs of sentences were evenly divided into two lists. Half of the participants received one list of the sentences, while the other half of the participants received the other list. No participants encountered both SRCs and ORCs in the same pairs during the fMRI experiment. Each participant read one list of sentences with 32 of each type overall (32 OM-SRCs and 32 OM-ORCs). In each list, all 64 sentences (32 in each type) were evenly divided into four sub-lists, each of which was employed in one fMRI run. Besides, as noted in the introduction, the data from our previous study [28] was also adopted for further comparison. The corresponding examples were also shown in Table 1C and 1D.

### Procedure

**Fig 1:**
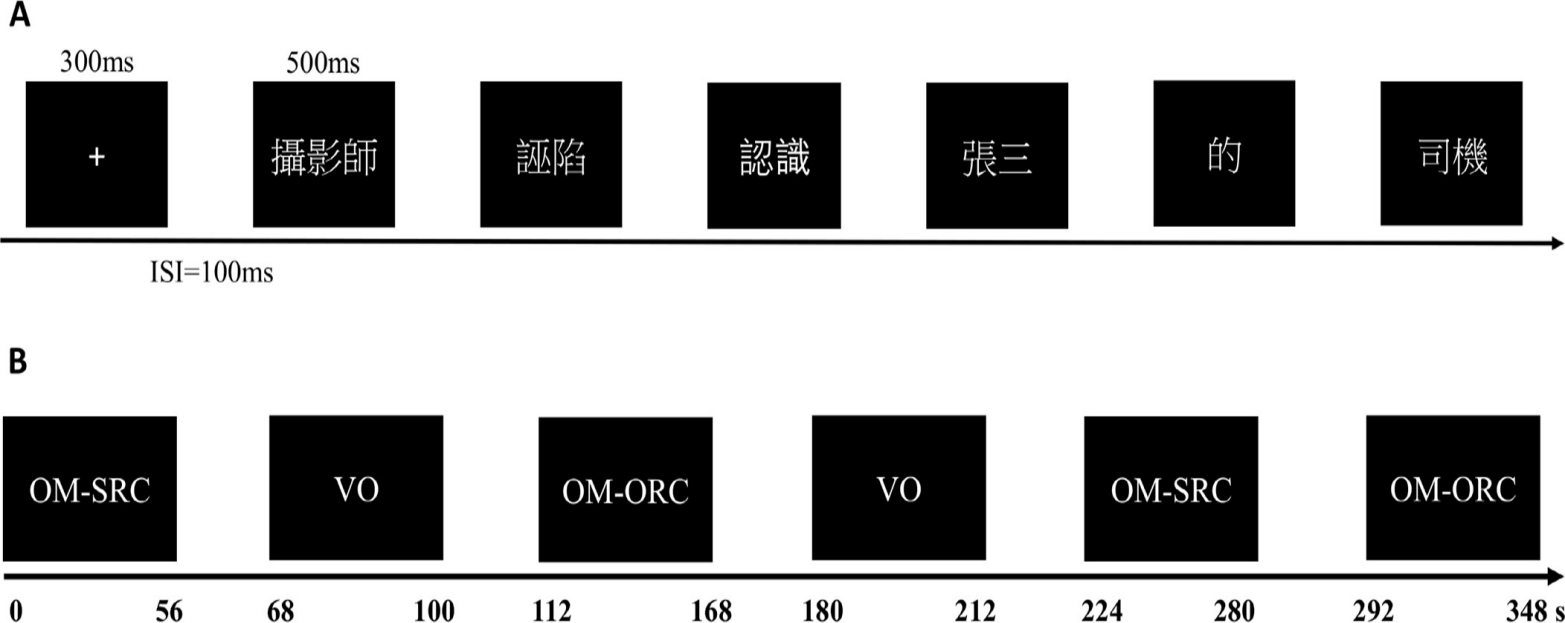
Experimental procedure. (A) The timeline of one sentence trial. Each trial starts with a fixation cross presented for 300 ms, followed by a 100 ms blank. Six frames are successively presented on the screen for 500 ms with 100 ms inter-stimulus interval. (B) The timeline of one run in the experiment. Each run contains six blocks, with two blocks of each type of sentences (OM-SRC and OM-ORC) and two blocks of the baseline condition (VO). The blocks in each run are in a pseudorandomized order. Note: VO = visual orientation; OM-SRC = object-modifying subject-extracted relative clause; OM-ORC = object-modifying object-extracted relative clause.

A mixed-trial fMRI design was employed in the present study, as reported in Xu et al. [28]. Simply, the main task was sentence comprehension, in which the stimuli were presented frame by frame at a steady rate to avoid possible eye movement during sentence reading. As illustrated in Fig 1A, each sentence started with a fixation cross for 300 ms, followed by a blank screen for 100 ms. Six frames were sequentially shown on the center of the screen for 500 ms with 100 ms inter-stimulus intervals. After the presentation of a whole target sentence, a comprehension question with the structure of ‘who did what to whom’ appeared on the screen to test whether the participants understand the meaning of the preceding sentence. Participants were required to make their responses by using the left or right thumb to indicate true or false, respectively. There was a 2s or 4s jitter before the beginning of the next sentence. In addition, the visual orientation (VO) task was used as a baseline condition to identify the brain areas related to sentence processing more than those to basic visual processing of stimuli. Specifically, participants were asked to make a same/different orientation judgment on visual stimuli that contained two rows of five arrows with the same or different orientations, one immediately above the other.

The whole experiment consisted of four runs with an equal length (6 minutes) and each run contained six blocks in a pseudorandomized order. Specifically, there were two blocks of the OM-SRC sentence reading (thus each block included four sentences), two blocks of the OM-ORC sentence reading (each also for four sentences), and two blocks of VO task as illustrated in Fig 1B. The order of the sentences in sub-lists was completely randomized across participants.

### Imaging protocol

The MRI images were acquired using a 3T scanner (MAGNETOM Skyra, Siemens, Erlangen, Germany) with a 64-channel whole-head coil located at National Cheng-Chi University, Taipei, Taiwan. The participants’ heads were immobilized with a vacuum-beam pad in the scanner. Functional, blood oxygenation level-dependent (BOLD) signals were acquired with a T2*-weighted echo planar imaging (EPI) sequence with the following parameters: slice thickness of 3.4 mm and no gap, in-plane resolution of 3.4375 × 3.4375 mm, and TR / TE / flip angle = 2000 ms / 70 ms / 77°. The field-of-view was 220 × 220 mm, and the acquisition matrix was 64 × 64. Thirty-four oblique-axial slices paralleled to the anterior commissure-posterior commissure (AC-PC) line were acquired to cover the whole brain. The first five volumes of each functional run were discarded for signal equilibrium, and a total of one hundred and eighty image volumes were acquired in each of the four runs. To obtain fine-grained localization information of the fMRI activity, a high-resolution anatomical brain image of each participant was obtained using a T1-weighted sequence (TR = 2530 ms / TE = 3.3 ms / flip angle = 7°, bandwidth = 200 Hz / pixel). This sequence used 192 sagittal slices to cover the whole brain and resulted in an isotropic spatial resolution of 1 × 1 ×1 mm^3^.

### Imaging data analysis

The fMRI data were processed with the Statistical Parametric Mapping package (SPM8) developed by the Wellcome Trust Center for Neuroimaging at University College London. The image data (including the target sentence and the probe question parts) with error responses were excluded from the analysis. Images of each participant were first adjusted for slice timing so that each slice’s time series was temporally aligned after correcting the time difference between the slice acquisitions in a volume. Then, the image volume series was realigned to the middle image volume of each run to correct for head movements with the 5^th^-order B-spline interpolation [31, 32]. A mean functional image volume was created for each participant and each run from the realigned image volumes and spatially normalized to an MNI (Montreal Neurological Institute) EPI template using a nonlinear affine transformation [33, 34]. The normalization parameters determined from the mean functional volume were then applied to the corresponding functional image volumes for each participant. Finally, the images were resampled to the voxel size of 2 mm x 2 mm x 2 mm and also smoothed with a Gaussian kernel of 8 mm full-width at half-maximum (FWHM).

An analytical, statistical design was constructed for each participant, using the general linear model (GLM) with the parameters consisting of the onsets of target sentence and visual orientation, convolved with a canonical hemodynamic response function and its temporal derivative [35, 36]. The six-degree-of-freedom realignment parameters were also included in the design matrix. The data was high-pass filtered temporally (128s cutoff) to remove low-frequency signal drifts. In the first-level analysis, we constructed for each participant two contrasts between each of the sentence comprehension conditions against the VO task (i.e., OM-SRC versus VO and OM-ORC versus VO). The brain activation at the first level was thresholded at *p* < 0.05, corrected for multiple comparisons using the family-wise error (FWE) [34]. The FWE was achieved by adjusting the significance level according to the formula, 1-(1-α_IT_)^c^, where *α*_*IT*_ is the alpha level for an individual test (e.g., 0.05) and *c* is the number of comparisons. Then, the ‘con’ or contrast images of the first-level analysis were utilized for the second-level group statistics using a one-sample T-test and the group results were visualized using the xjView toolbox (http://www.alivelearn.net/xjview) to identify selected regions of interests (ROIs). As noted in the introduction, the LIFG and the LSTG were two main ROIs in this study. However, only the LIFG was preserved from the direct contrast between the SRCs and the ORCs, we thus directly extracted the peak information of the LIFG (x = -52, y = 6, z = 10) from the direct contrast result, but extracted the peak information of the LSTG (x = -56, y = -46, z = 4) from the whole-brain activation. Afterwards, we used MarsBaR (http://marsbar.sourceforge.net/) in SPM8 to extract the adjusted BOLD time courses of those ROIs (i.e., the LIFG and the LSTG) with a 5-mm radius for each participant [37].

### Granger causality analysis

The Granger causality (GC) is based on the delayed version of the time courses for deriving the temporal dependence [38]. This is different from the GLM that has been used to compute the multi-regression model of the fMRI time series data at each of the single voxel against a combination of reference functions in the design matrix. As a result, after the activation analysis using the GLM model to identify the brain regions significantly involved in sentence comprehension task, it should be of interest to compute the GC of those significantly activated brain areas, such as the LIFG and the LSTG in this study, to investigate the directed interactions between them. We refer the reader to Xu et al. [28] for the technical details of the GC. Simply, we first concatenated the trial time courses of the same RC conditions (OM-SRCs and OM-ORCs) from all four runs in order at the single-voxel level from each ROI (i.e., the LIFG and the LSTG). Then, the conditional time courses (OM-SRCs and OM-ORCs) for further computation were obtained by averaging the time courses of all voxels within each of the ROIs. Afterwards, the GC was computed using the bivariate autoregression (AR) model on the time courses from each pair of those two ROIs under each of the two conditions. Specifically, we calculated the residual variance using the bivariate AR model with the optimal lag number determined using the Bayesian information criterion for each time course separately. The F values of the differences between the residual variances with and without the other time course included in the bivariate AR model were computed and tested using a significance level of *p* < 0.01. Finally, the binomial test with a significance level of *p* < 0.05 was used to further summarize the group results regarding the directional connections between the LIFG and the LSTG [35]. Moreover, to validate the observed connectivity between those two brain areas, we further tested the significance of the strength difference between the GC results (the F values) using a Wilcoxon Signed Rank test [39].

## Results

### Behavioral results

The accuracy and reaction time (RT) of the responses to the comprehension questions were recorded. Only the RTs of accurate responses and within two standard deviations (SDs) around the mean were included for further analysis. For the OM-RC sentences, the accuracy (mean ± SD) across participants was 87.91 ± 7.96%. The accuracy of the OM-SRC sentences (85.69 ± 10.12%) was numerically lower than that of the OM-ORC sentences (90.13 ± 4.20%) (*t* (18) = -1.874, *p* = 0.077). As for RT (mean ± SD), the contrast between the OM-SRC (1308.66 ± 241.77 ms) and OM-ORC (1292.2 ± 200.48 ms) conditions did not result in any significant difference (*t* (18) = 0.262, *p* = 0.797). For the SM-RC sentences, the overall comprehension accuracy (mean ± SD) across participants was 94.33 ± 3.90%, with an accuracy of 94.57 ± 3.10% for the SM-SRC sentences and an accuracy of 94.08 ± 4.65% for the SM-ORC sentences. No significant difference was found between them (*t* (18) = 0.389, *p* = 0.702). With regard to RT (mean ± SD), there was also no significant difference (*t* (18) = - 0.060, *p* = 0.953) between the SM-SRC (1137.88 ± 194.55 ms) and SM-ORC (1139.17 ± 161.62 ms) sentences.

**Fig 2:**
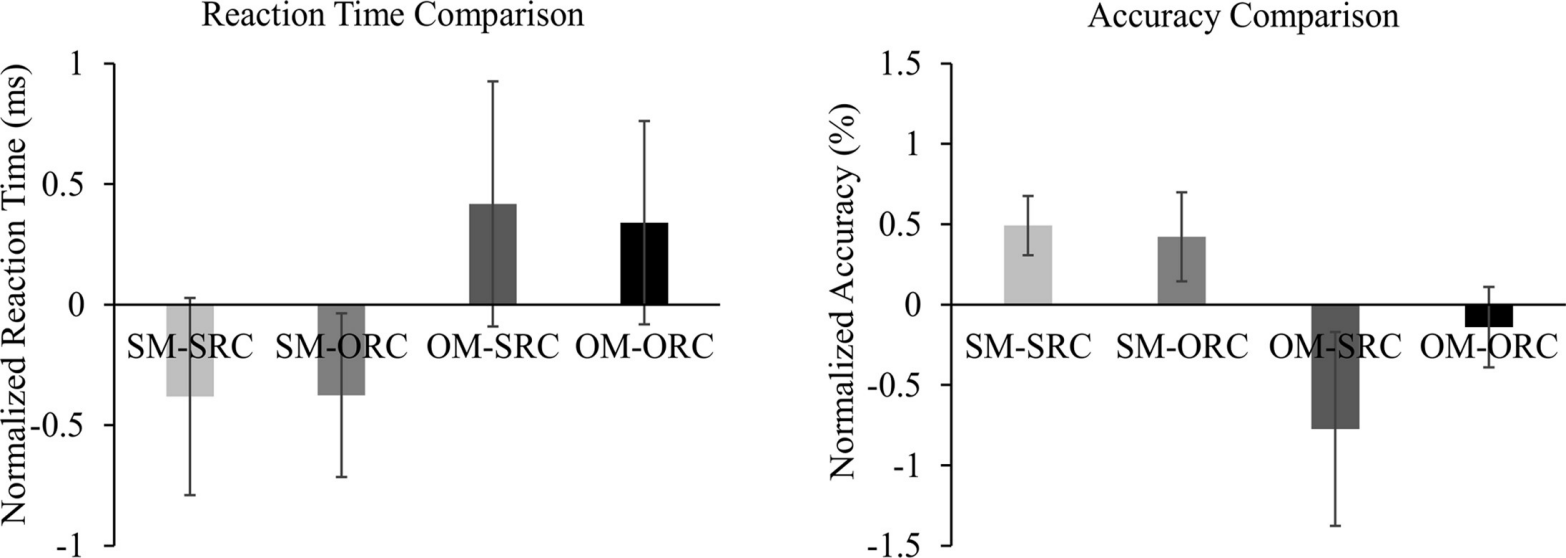
The comparison of the comprehension performance in SM-SRC, SM-ORC, OM-SRC, and OM-ORC sentences. The results show that the OM-RC sentences are more difficult to process than the SM-RC sentences. Among all sentences, OM-SRCs seems to be the most difficult one to comprehend, followed by OM-ORCs, SM-SRCs, and SM-ORCs.

To see how the modifying position and extraction position affect the comprehension performance, we first normalized the behavioral data collected from the SM-RC and OM-RC experimental trials by converting the measures to z-scores. The results are depicted in Fig 2. Then, we conducted a two-way ANOVA and a non-parametric test to examine the significance of the difference between different types of sentences. The results showed that with regard to RT, there was a significant main effect in modifying position (*F* (1, 18) = 6.870, *p* = 0.017, *η*_*p*_^*2*^ = 0.276), with OM-RCs being slower to process overall than SM-RCs, but not a significant main effect in extraction position (*F* (1, 18) = 0.059, *p* = 0.812, *η*_*p*_^*2*^ = 0.003). Additionally, no significant interaction between modifying position and extraction position was found (*F* (1, 18) = 0.064, *p* = 0.803, *η*_*p*_^*2*^ = 0.004). As for the accuracy, the non-parametric Friedman test indicated that there was a significant difference in modifying position (*χ*^*2*^ (3) = 17.183 *p* < 0.001, with a mean rank of 3.16 for SM-SRCs, 3.03 for SM-ORCs, 1.82 for OM-SRCs and 2.00 for OM-ORCs). A Wilcoxon Signed Ranks test with Bonferroni correction was further conducted to test pairwise comparison. The results showed that the accuracy of OM-SRCs was significantly lower compared to SM-SRCs (*Z* = -3.056, *p* = 0.002) and SM-ORCs (*Z* = -2.638, *p* = 0.008). Besides, the accuracy of OM-ORCs was also statistically lower than that of SM-SRCs (*Z* = -3.345, *p* = 0.001) and SM-ORCs (*Z* = -2.795, *p* = 0.005). However, there were no significant differences between SM-SRCs and SM-ORCs (*Z* = -0.370, *p* = 0.711) and between OM-SRCs and OM-ORCs (*Z* = -1.393, *p* = 0.164).

Therefore, the OM-RC sentences were found to be significantly harder to comprehend than the SM-RC sentences because of the lower accuracy and also slower RT in the OM-RC sentences. Besides, regardless of the modifying position, SRCs seemed to be comparable with ORCs, although the sentences with ORCs showed some numerical advantage (relatively higher accuracy and faster RT) than the sentences with SRCs.

### Imaging results

#### Whole-brain analysis

**Fig 3:**
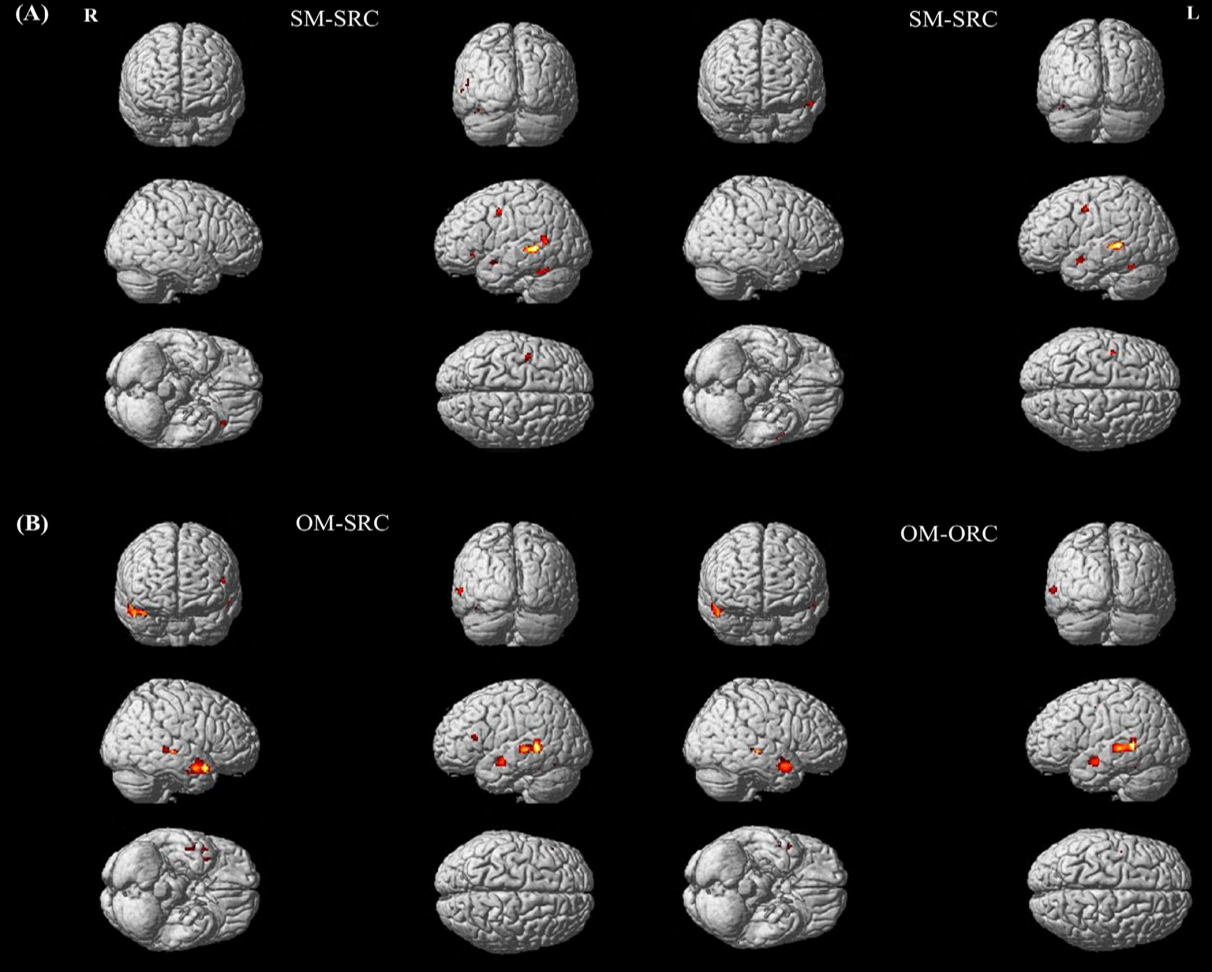
Volume rendering of SM-SRC, SM-ORC, OM-SRC and OM-ORC sentences. The similar bilateral activation patterns are identified in response to the processing of the SRC and the ORC, regardless of the modifying position. The results show that (A) for the SM-RC sentences, only a left-lateralized frontotemporal neural network is involved; whereas (B) for the OM-RC sentences, besides the typical left-hemispheric brain areas, the right superior temporal gyrus (RSTG) is also recruited in sentence processing.

As shown in Fig 3A, during the processing of the SM-RC sentences, only a left-lateralized frontotemporal neural network was identified. On the other hand, for the OM-RC sentences as displayed in Fig 3B, in addition to the typical left neural network as observed in the SM-RCs, the right superior temporal gyrus (RSTG) was also shown to be involved in sentence processing after contrasting each type of sentences with the VO task. Besides, although the similar activation pattern was found in both the OM-SRC and the OM-ORC sentences, the extent of the activation in activated brain areas was numerically larger in the OM-SRC than the OM-ORC sentences as indicated in Table 2. Table 2 summarizes the coordinates of peak activation in the regions that are activated at the significance level of *p* < 0.05 (corrected by FWE) for each type of sentences in contrast with the control VO task.

**Table 2:** Locus and extent of peak activation in brain regions during sentence comprehension.

				MNI coordinate (mm)					
Scan	Anatomical Region	BA	Voxels	x	y	z	T	p	Hemisphere
OM-SRC	Superior temporal gyrus	22	282	-56	-46	4	10.28	<0.001	L
	Superior temporal gyrus	22	270	42	10	-22	10.14	<0.001	R
	Middle temporal gyrus	21	78	-52	-6	-14	8.43	0.004	L
	Middle temporal gyrus	21	35	58	-24	-2	9.29	0.001	R
	Fusiform Gyrus	37	10	-40	-48	-20	7.71	0.012	L
	Inferior frontal gyrus	44/45	21	-50	24	14	8.01	0.008	L
OM-ORC	Superior temporal gyrus	22	300	-56	-46	4	9.31	0.001	L
	Superior temporal gyrus	22	47	50	-30	0	6.77	0.040	R
	Middle Temporal gyrus	21	83	-54	-2	-14	8.14	0.005	L
	Middle Temporal gyrus	21	47	58	-24	-4	9.73	0.001	R
	precentral gyrus	6	1	-44	-4	48	6.74	0.042	L
	Fusiform gyrus	37	3	-40	-46	-20	6.89	0.034	L
SM-SRC	Superior temporal gyrus	22	147	-54	-42	2	6.55	<0.001	L
	Temporal pole	38	10	-50	6	-14	5.43	0.002	L
	Precentral gyrus	6	25	-40	-2	44	5.38	0.003	L
	Fusiform Gyrus	37	75	-40	-56	-22	5.34	0.003	L
	Inferior frontal gyrus	47	12	-36	28	-6	5.02	0.013	L
SM-ORC	Superior temporal gyrus	22	120	-54	-40	2	6.04	<0.001	L
	precentral gyrus	6	23	-40	-4	44	5.40	0.002	L
	Middle temporal gyrus	21	27	-56	0	-16	5.25	0.005	L
	Fusiform Gyrus	37	28	-40	-58	-20	5.07	0.011	L
	Posterior cingulate cortex	23	12	-2	-58	18	5.02	0.012	L

The contrasts are significant at family-wise error (FWE) rate threshold of *p* < .05. Coordinates are reported in MNI space and refer to the peak Z scores for each region. OM-SRC = object modifying subject-extracted relative clause; OM-ORC = object modifying object-extracted relative clause; SM-SRC = Subject modifying subject-extracted relative clause; SM-ORC = subject modifying object-extracted relative clause; BA = brodmann area; L = left; R = right.

**Fig 4:**
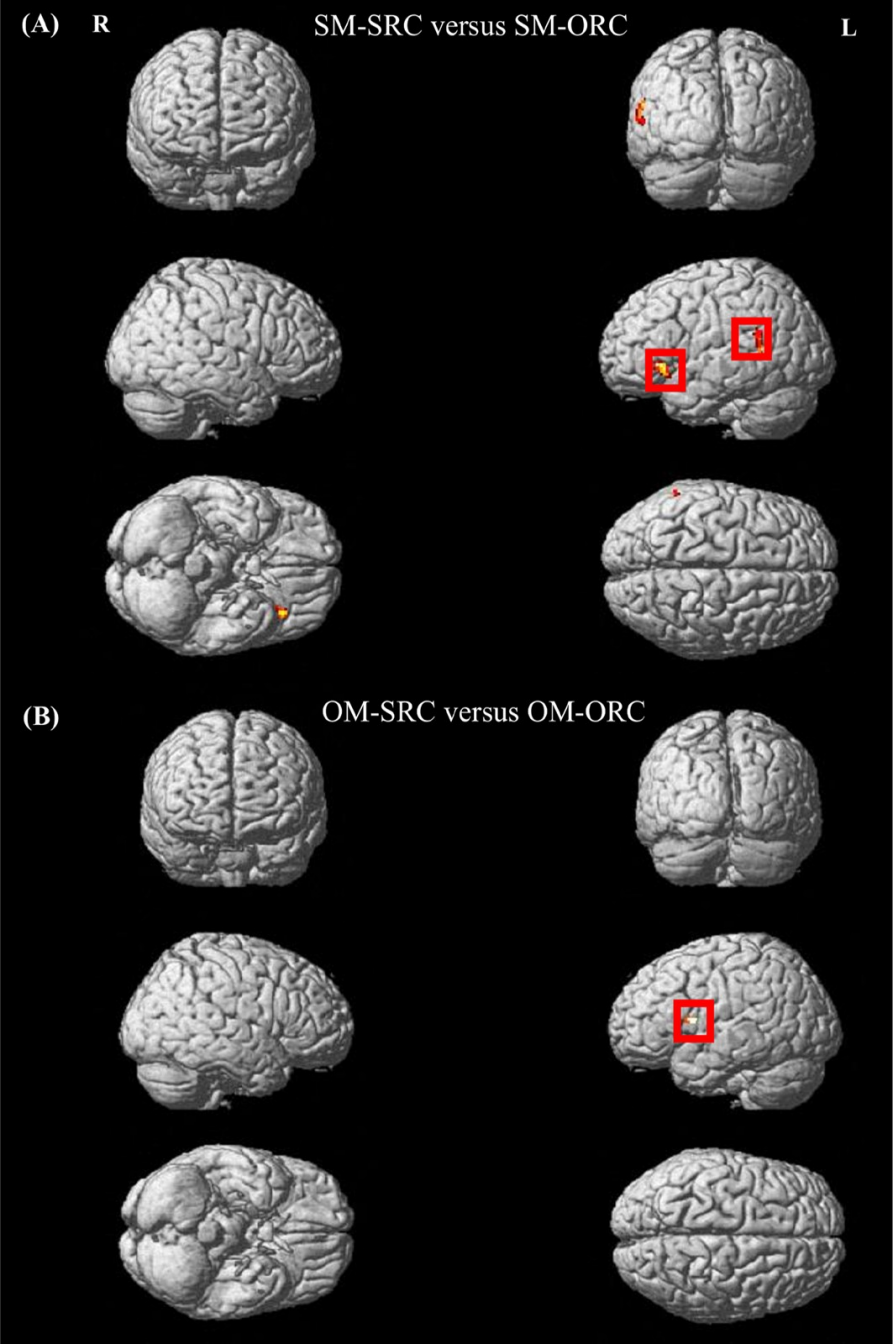
Volume rendering of the contrast between the SRCs and the ORCs in different modifying positions. The results indicate that (A) greater activation in the LIFG and the LSTG is significantly involved in the processing of the SM-SRC sentences compared to the SM-ORC sentences; (B) enhanced activation in the LIFG is significantly evoked by the OM-SRC sentences rather than the OM-ORC sentences.

We further conducted a direct contrast analysis on each pair of the SM-RC sentences as well as each pair of the OM-RC sentences to highlight the brain activation associated with sentence comprehension. As shown in Fig 4, greater activation in the LIFG and the LSTG was significantly evoked during reading the SM-SRC sentences than during reading the SM-ORC sentences. As for the OM-RC sentences, the OM-SRC sentences significantly induced higher activation in the LIFG for sentence comprehension than the OM-ORC sentences. Therefore, these results together illustrated that regardless of the modifying position, greater brain activation, hence higher processing demands, was required in the processing of the SRC sentences than in processing the ORC sentences.

#### Granger causality (GC) results

The GC results showed that the LIFG significantly Granger-caused the LSTG during the comprehension of the SM-SRC (*p* = 0.0318, binomial test) rather than the SM-ORC sentences (*p* = 0.9682, binomial test). On the other hand, the GC from the LSTG to the LIFG was far from significance in both conditions (SM-SRC: *p* = 0.6762; SM-ORC: *p* = 0.6762). Moreover, the Wilcoxon Signed Ranks Test indicated that the strength of the GC from the LIFG to the LSTG in the SM-SRC condition was significantly stronger than that in the SM-ORC condition (*Z* = 2.575, *p* = 0.008); by contrast, the strength of the GC from the LSTG to the LIFG revealed no significant difference between both conditions (*Z* = 1.288, *p* = 0.210). Besides, during the processing of the SM-SRC sentences, the connection from the LIFG to the LSTG was found to be significantly stronger than that in the opposite direction of these two brain areas (*Z* = 2.334, *p* = 0.018).

On the other hand, for the OM-RC sentences, in view of the important role of the LSTG in the processing of RC sentences (see Table 2), we also included the LSTG with the peak location at (x = -56, y = -46, z = 4) for the GC analysis. The binomial test found that the LSTG significantly Granger-caused the LIFG in both conditions (OM-SRC, *p* = 0.0318; OM-ORC, *p* = 0.0318), whereas the GC from the LIFG to the LSTG was far from significant in both conditions (OM-SRC, *p* = 0.9904; OM-ORC, *p* = 0.9682). On the other hand, the Wilcoxon Signed Ranks Test showed that there was no significant difference in the connectivity strength between the OM-SRCs and OM-ORCs (the connection from the LSTG to the LIFG, *Z* = 0.523, *p* = 0.623; the connection from the LIFG to the LSTG, *Z* = 1.046, *p* = 0.312). However, the strength of the connectivity from the LSTG to the LIFG was significantly stronger than that of the opposite direction during the processing of the OM-SRCs (*Z* = 2.535, *p* = 0.009) and also the OM-ORCs (*Z* = 2.415, *p* = 0.014). Therefore, the feedback mechanism from the LSTG to the LSTG might play primary role in the comprehension of the OM-RC sentences.

#### Brain activation attunes to different sentence complexity

To explore how the modifying position and extraction position influence regional brain activation during sentence processing, we conducted a two-way ANOVA on the data from the SM-RC and OM-RC sentences to test the significance of the activation difference in the LIFG and the LSTG. The results showed that for the activation in the LIFG, there was a significant main effect in modifying position (*F* (1, 18) = 32.390, *p* < 0.001, *η*_*p*_^*2*^ = 0.643), with enhanced activation for the comprehension of the OM-RC compared to the SM-RC sentences. There was also a significant main effect in extraction position (*F* (1, 18) = 4.399, *p* < 0.05, *η*_*p*_^*2*^ = 0.196), with the sentences with SRCs inducing greater activation in the LIFG overall than the sentences with ORCs. However, no significant interaction between modifying position and extraction position was found (*F* (1, 18) = 2.538, *p* = 0.129, *η*_*p*_^*2*^ = 0.124). On the other hand, for the activation in the LSTG, there were significant main effects in modifying position (*F* (1, 18) = 19.528, *p* < 0.001, *η*_*p*_^*2*^ = 0.520) and extraction position (*F* (1, 18) = 16.607, *p* = 0.001, *η*_*p*_^*2*^ = 0.480). Specifically, greater activation in the LSTG was significantly recruited during the comprehension of the OM-RC than the SM-RC sentences. The sentences with SRCs tend to involve greater activation in the LSTG compared to the sentences with ORCs. In addition, there was a significant interaction between modifying position and extraction position (*F* (1, 18) = 17.836, *p* = 0.001, *η*_*p*_^*2*^ = 0.498). Post-hoc analysis with Bonferroni correction further showed that a significant activation difference in the LSTG was only observed when the RC was located at the subject-modifying position (*p* < 0.001) rather than the object-modifying position (*p* = 0.675).

**Fig 5:**
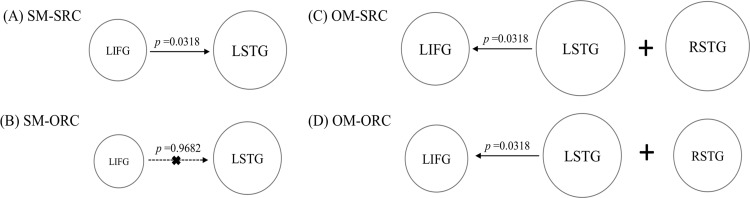
The simplified graph for the processing mechanism involved in the RC sentences with different levels of processing difficulty. The results illustrate A) The activation of the LIFG and the LSTG, as well as the connectivity from the LIFG to the LSTG, is involved in the processing of the SM-SRCs; B) No evident functional communication between the LIFG and the LSTG is found in the reading of the SM-ORCs, although relatively less activation in the LIFG and the LSTG is still needed; C) The activation in the LIFG and also in the LSTG and its right homolog, as well as the effective connectivity from the LSTG to the LIFG, together facilitate the processing of the OM-SRCs; D) Compared with the OM-SRCs, the similar neural mechanism is involved in the reading of the OM-ORCs, although the activation of the LIFG is significantly less in the OM-ORCs compared to that in the OM-SRCs.

Regarding the information flow between the LIFG and the LSTG, the results showed that the connectivity from the LIFG to the LSTG rather than the opposite direction was significantly involved in the reading of the SM-RCs but only associated with the SM-SRCs. On the other hand, the feedback connection from the LSTG to the LIFG was engaged in the comprehension of both the OM-SRC and OM-ORC sentences (see Fig 5). To be noted, because we primarily aimed to explore the connectivity pattern of the left-hemispheric syntax-sensitive regions, in the present study, we only considered the GCs between the LIFG and the LSTG. Furthermore, a non-parametric Friedman test was used to examine whether and how the complexity effect influences the connectivity strength between the LIFG and the LSTG. The results showed that there was a significant difference in the strength of the connectivity from the LIFG to the LSTG across sentences (*χ*^*2*^ (3) = 17.1337, *p* = 0.001, with a mean rank of 3.53 for the SM-SRCs, 2.21 for the SM-ORCs, 1.89 for the OM-SRCs and 2.37 for the OM-ORCs). A Wilcoxon Signed Ranks test with Bonferroni correction further indicated that stronger connectivity from the LIFG to the LSTG was significantly involved in the processing of the SM-SRCs in comparison to the SM-ORCs (*Z* = 2.575, *p* = 0.010). Meanwhile, the strength of the connectivity from the LIFG to the LSTG did not result in any significant difference between the OM-SRCs and the OM-ORCs (*Z* = 1.046, *p* = 0.295). On the other hand, the strength of the connectivity from the LSTG to the LIFG did not result in any significant differences across sentences (*χ*^*2*^ (3) = 4.074, *p* = 0.254).

Therefore, the abovementioned brain activations, as well as the connectivity findings, might indicate different neural mechanisms involved in the processing of RC sentences with different complexity (as depicted in Fig 5). Additionally, although the extent of the activation strength in the RSTG was only numerally greater in the OM-SRCs compared to the OM-ORCs, it might somehow reflect the extent of the processing loadings involved in sentence processing. Thus, the RSTG was also included in the simplified graph below.

## Discussion

The present data clearly depicted the neural correlates underlying the processing of Chinese OM-RC sentences. Together with the findings in the SM-RC study [28], the conclusion can be safely made that the processing asymmetry between Chinese SRCs and ORCs is mainly due to the word order effect. In other words, regardless of the modifying position, Chinese SRCs with non-canonical word order were consistently more difficult to process than the ORCs, as reflected in increased brain activation in the LIFG and the LSTG and the directional connectivity between them. Moreover, the present 2 × 2 factorial analyses revealed that the BOLD activation in the LIFG and the LSTG dynamically attuned to different processing difficulty, which was manipulated by different modifying and extraction positions. Specifically, the activation strength in the LIFG was shown to be sensitive to the extraction position effect; that is, the SRCs significantly elicited greater activation in the LIFG than the ORCs, regardless of the modifying position (subject- or object-modifying). Regarding the activation strength in the LSTG, the extraction position effect was only observed when the RC was at the subject-modifying rather than the object-modifying position. Furthermore, the directional connectivity between the LIFG and the LSTG appeared to be associated with the modifying position effect. To be specific, when the RC was at the subject-modifying position, the connectivity from the LIFG to the LSTG was activated, especially for the SM-SRCs; whereas when the RC was at the object-modifying position, it was the connectivity from the LSTG to the LIFG that might take place in sentence processing.

As noted in the introduction, the processing of the SM-SRCs was modulated by the information flow from the LIFG to the LSTG to support word-order analysis. Different from the SM-RC sentences, both types of the OM-RC sentences are initiated by the same noun-verb sequence, followed by a verb-noun-relativizer sequence in an SRC or by a noun-verb-relativizer sequence in an ORC. Due to incremental nature of sentence processing [40–42], stored information in the LSTG appeared to be first accessed before reaching the RC, implying that the driving force in the processing of this type of complex sentences might be from the LSTG. Afterwards, the lexico-semantic information retrieved from the LSTG was conveyed to the LIFG for constructing argument hierarchy by ordering the arguments. Due to the noncanonical word order, greater activation and stronger connectivity were elicited for processing the SRC structure than the ORC structure. In this scenario, sentence processing seems start from analysis of verbs and their argument structure subcategorizations rather than structure building based on word order [27]. It should be noted that we are fully aware that the network we present is far from complete. Some brain areas in addition to the LIFG and the LSTG may also play important roles in processing and exchanging information. In the present study, our analyses are constrained in the investigation of the information flow between two crucial regions involved in the processing of RC sentences. Moreover, our results do not completely exclude the possibility of bi-directional connectivity between the LIFG and the LSTG. Instead, we propose that the directional connectivity between the LIFG and the LSTG may be modulated by different types of complex sentences as shown in the present results.

On the other hand, in addition to the involvement of the LIFG and the LSTG that has been commonly identified as forming a ‘core syntax’ network [17, 43–46] for sentence comprehension, the right-hemispheric activation, especially the activation in the RSTG, appeared to participate in the processing of the OM-RC sentences. Previously, few studies reported the right-hemispheric syntax-sensitive brain regions during the processing of RC sentences, and some studies even claimed that the right hemisphere was not associated with the processing of complex syntactic information, particularly in right-handers [47]. It is rather known that a bilateral organization of the language networks supports many different aspects of linguistic processing, such as the acquisition of phonological presentations [48–51], memory of new words by using semantic association [52], and also the processing of prosodic features of speech [8, 53]. As shown in the current findings, such bilateral activation might also support the processing of syntactic information. Considering the results are mainly from the BOLD signal subtraction analyses and the relatively weak evidence is for the involvement of the right-hemispheric activation, further study will be needed to test this assumption.

In sum, the present findings indicate the relative specialization but extensive collaboration involved in the LIFG and the LSTG during the processing of Chinese RC sentences. The brain network seems to be organized dynamically according to different levels of processing difficulty in the task at the process. Therefore, the optimal strategy to recruit participating brain areas and to introduce the information flow among them is proposed to be dynamically allocated to comprehend sentences with different levels of complexity.

## Supporting information

S1 File (PDF)
