# Supplementary material for: Brain connectivity in the left frontotemporal network dynamically modulated by processing difficulty: Evidence from Chinese relative clauses
Source: PLoS One. 2020 Apr 9;15(4):e0230666. doi: 10.1371/journal.pone.0230666 (PMC7144993; doi:10.1371/journal.pone.0230666)
Supplement: S1 File — (PDF) [file pone.0230666.s001.pdf]

Brain and Language 200 (2020) 104712

Contents lists available at ScienceDirect

Brain and Language

journal homepage: www.elsevier.com/locate/b&l

Enhanced left inferior frontal to left superior temporal effective connectivity for complex sentence comprehension: fMRI evidence from Chinese relative clause processing

Kunyu Xu[a], Denise H. Wu[a], Jeng-Ren Duann[a,b,*]

[a] Institute of Cognitive Neuroscience, National Central University, Taoyuan 32001, Taiwan
[b] Institute for Neural Computation, University of California San Diego, La Jolla, CA 92093, USA

ARTICLE INFO

Keywords:
Relative clause
Sentence processing
Sentence complexity
Effective connectivity

ABSTRACT

Previous studies investigating the processing of complex sentences have demonstrated the involvement of the left inferior frontal gyrus (LIFG) and left superior temporal gyrus (LSTG), which might subserve ordering and storage of linguistic components, respectively, for sentence comprehension. However, how these brain regions are interconnected, especially during the processing of Chinese sentences, need to be further explored. In this study, the neural network supporting the comprehension of Chinese relative clause was identified. Both the LIFG and LSTG exhibited higher activation in processing subject-extracted relative clauses (SRCs) than object-extracted relative clauses (ORCs). Moreover, a Granger causality analysis revealed that the effective connectivity from the LIFG to LSTG was significant only when participants read Chinese SRCs, which were argued to be more difficult than ORCs. Contrary to the observations of an SRC advantage in most other languages, the present results provide clear neuroimaging evidence for an ORC advantage in Chinese.

1. Introduction

Relative clause (RC), which can be used to produce sentences with the embedded structure in natural languages, has attracted much attention in psycholinguistics. It has been claimed that the capacity to process recursively embedded structure is unique and critical for human beings in the generation and understanding of an unlimited amount of sentences (e.g., Friederici, Bahlmann, Heim, Schubotz, & Anwander, 2006). To achieve such processing, the human brain needs to access information from words in a sequence and then store and order the accessed information as required to understand the meaning of 'who is doing what to whom' (e.g., Fiebach, Schlesewsky, & Friederici, 2001; Kuhnke, Meyer, Friederici, & Hartwigsen, 2017; Meyer, Obleser, Anwander, & Friederici, 2012; Phillips, Kazanina, & Abada, 2005; Ueno & Kluender, 2003). Different word sequence arrangements in a sentence may create different relations between dependencies, which result in different loadings of various mental operations during the storage and ordering processes (Caplan, Stanczak, & Waters, 2008; Cooke et al., 2002; Gibson, 1998; Grodner & Gibson, 2005; Just, Carpenter, Keller, Eddy, & Thulborn, 1996; Stowe et al., 1998).

There are mainly two types of RC sentences, namely, the sentences with a subject-extracted relative clause (SRC) (e.g., the reporter [*that attacked the senator*] admitted the error) and those with an object-extracted relative clause (ORC) (e.g., the reporter [*that the senator attacked]* admitted the error). Those SRC and ORC sentences share the equivalent sentence length and lexical content but different word order in the RC to present different relations between the verb and its arguments. Many studies have indicated that different word orders in SRCs and ORCs result in different levels of processing difficulty (e.g., Caplan et al., 2008; Cooke et al., 2002; Gibson, 1998; Grodner & Gibson, 2005; Just et al., 1996; Stowe et al., 1998). According to the Dependency Locality Theory (DLT) proposed by Gibson (1998), such difference in processing difficulty is primarily due to different working memory loads in maintaining and integrating the filler-gap dependencies with various distances (e.g., Gibson, 1998; Hsiao & Gibson, 2003; Just & Carpenter, 1992; King & Just, 1991). For example, in English, SRCs have been found to be easier to comprehend than ORCs (e.g., Caplan et al., 2008; Constable et al., 2004; Friederici, Makuuchi, & Bahlmann, 2009; Gibson, 1998; Grodner & Gibson, 2005; Just et al., 1996; King & Just, 1991; King & Kutas, 1995; Stromswold, Caplan, Alpert, & Rauch, 1996; Traxler, Morris, & Seely, 2002). Because English ORCs have noncanonical (i.e., Object-Subject-Verb) word order, which demands non-local integration across a longer filler-gap distance, a greater processing cost is required in the storage and ordering processes for the comprehension of English ORCs compared to that for the

* Corresponding author.

https://doi.org/10.1016/j.bandl.2019.104712
Received 10 December 2018; Received in revised form 18 September 2019; Accepted 24 October 2019
0093-934X/ © 2019 Published by Elsevier Inc.

comprehension of English SRCs with canonical word order (i.e., Subject-Verb-Object).

Although a clear preference for SRCs has been established in most languages, findings against an SRC preference in some languages, such as Basque (Carreiras, Duñabeitia, Vergara, Cruz-Pavía, & Laka, 2010) and Chinese (e.g., Chen, Ning, Bi, & Dunlap, 2008; Gibson & Wu, 2013; Hsiao & Gibson, 2003; Qiao, Shen, & Forster, 2012; Sung, Cha, Tu, Wu, & Lin, 2016), have been reported in the literature. Even within the same language, such as Chinese, the preference for SRCs (e.g., Vasishth, Chen, Li, & Guo, 2013) or ORCs (as cited above) is still a matter of debate. It is of theoretical importance to further examine the preference of Chinese RC processing and to shed light on the underlying factors for RC processing preference across languages.

In previous literature, two language-related brain regions, the left inferior frontal gyrus (LIFG) and the left superior temporal gyrus (LSTG), have been considered as prime candidate areas underlying the processing of RC sentences. Moreover, the strength of fMRI activation in these two regions has been commonly used as a neuroimaging marker to reflect the processing asymmetry between SRCs and ORCs (e.g., Friederici, 2011; Just et al., 1996; Sakai, 2005; Santi & Grodzinsky, 2010; Skeide, Brauer, & Friederici, 2016). Both the LIFG and the LSTG have been shown to be involved in sentence processing, but their functional roles might be distinct (e.g., Friederici, Rüschemeyer, Hahne, & Fiebach, 2003; Kuhnke et al., 2017; Meyer et al., 2012; Skeide, Brauer, & Friederici, 2014). Specifically, the LIFG has been claimed to support the ability to learn syntactic rules (Musso et al., 2003; Opitz & Friederici, 2004; Tettamanti et al., 2002) or take a subordinate role in syntactic parsing, such as ordering for the establishment of argument order (Ben-Shachar, Hendler, Kahn, Ben-Bashat, & Grodzinsky, 2003; Ben-Shachar, Palti, & Grodzinsky, 2004; Bornkessel, Zysset, Friederici, Cramon, & Schlesewsky, 2005; Fiebach et al., 2001; Friederici, 2011; Friederici et al., 2003; Newman, Pancheva, Ozawa, Neville, & Ullman, 2001). For example, increased activation in the LIFG has been commonly found during the processing of the complex sentences that require a hierarchical ordering due to an atypical word order, compared to the sentences with adjacent elements in canonical order (Ben-Shachar et al., 2003; Bornkessel, Zysset, Friederici, Cramon, & v., & Schlesewsky, M. , 2005; Friederici et al., 2006; Meyer et al., 2012). Kuhnke et al. (2017) further provided evidence in support of the causal role of the LIFG for the ordering process during sentence processing by using repetitive transcranial magnetic stimulations (rTMS). Although some studies have suggested a different role of the LIFG to support subvocal rehearsal of stored information (Rogalsky, Matchin, & Hickok, 2008; Rogalsky & Hickok, 2010) rather than underlying the process of ordering, clinical evidence has indicated that the ordering process in sentence comprehension could be selectively impaired while the active subvocal rehearsal remains intact (Caplan & Waters, 1999; Waters & Caplan, 1996).

Aside from the LIFG, the LSTG is another classic brain area that plays a key role in sentence comprehension. The LSTG has been shown to mediate the integration of semantic and syntactic information (Friederici, 2011; Friederici et al., 2003; Grodzinsky & Friederici, 2006; Newman et al., 2001) or be engaged in the short-term storage of argument-verb relations during the processing of complex sentences (Bornkessel et al., 2005; Friederici, 2011; Grodzinsky & Friederici, 2006; Kim et al., 2002; Newman et al., 2001; Novais-Santos et al., 2007; Owen, McMillan, Laird, & Bullmore, 2005; Smith & Jonides, 1998). For instance, Keller, Carpenter, and Just (2001) have found that greater activation in the LSTG is significantly elicited during the comprehension of English ORC sentences with a longer filler-gap distance compared to English SRC sentences with a relatively short filler-gap distance. The similar finding has also been reported in Friederici et al. (2009)'s study using German sentences. A clinical study of stroke patients further claimed that the integrity of the LSTG was closely correlated with the patients' digit span scores and also their comprehension of spoken sentences, supporting the contribution of short-term memory to sentence processing (Leff et al., 2009). Recent evidence has consistently shown increased activation in the LSTG during the comprehension of sentences with a longer argument-verb distance in comparison to those with a shorter distance, with such increased activation being thought to reflect enhanced storage demands (Kuhnke et al., 2017; Meyer et al., 2012). Together, the LSTG might be closely associated with the storage process that was required to retain phrases intervening between critical arguments and the verb for sentence comprehension. Based on the accumulated research, the operations of storage and ordering during the processing of argument-verb dependency are supported by behavioral and event-related brain potential evidence (e.g., Clahsen & Featherston, 1999; Fiebach et al., 2001; Nakano, Felser, & Clahsen, 2002; Ueno & Kluender, 2003), as well as by the neuroimaging findings showing that the LIFG and the LSTG subserve the ordering and storage processes, respectively.

Similar to the comprehension of English RC sentences, the storage and ordering processes are both needed to determine who is doing what to whom in the processing of Chinese SRC and ORC sentences. According to the DLT (Gibson, 1998), a greater processing cost for storage and ordering of argument-verb relations is needed in the comprehension of Chinese SRC sentences, whose word order in the RC (Object-Verb-Subject) is non-canonical, compared to that in Chinese ORC sentences with canonical word order in the RC. Specifically, during the comprehension of Chinese SRC sentences (e.g., gōngjī Zhāngsān de sījī wěifǎnle guīdìng '*The driver who attacked Zhangsan has violated the rules*', as shown in Example A below), three syntactic heads before the head noun 'sījī' (i.e., 'gōngjī', 'Zhāngsān' and 'de') need to be stored in working memory and then be integrated across a long filler-gap distance to establish the argument hierarchy for the understanding of 'who did what to whom'. On the other hand, during the comprehension of Chinese ORC sentences (e.g., Zhāngsān gōngjī de sījī wěifǎnle guīdìng '*The driver who Zhangsan attacked has violated the rules*', as shown in Example B), there is only one predicted head required during sentence reading and all the arguments are incrementally integrated across a relatively short filler-gap distance. Thus, Chinese SRC sentences are predicted to be more difficult to comprehend than Chinese ORC sentences. Therefore, in light of the preceding discussion, the first aim of this study was to investigate whether the hemodynamic responses in previously identified brain regions during the processing of RC sentences could provide direct neuroimaging evidence for an ORC preference in Chinese. Specifically, based on previous neuroimaging research on the RC sentence processing in other languages (due to a lack of any prior research in Chinese), different involvements of the LIFG and the LSTG, which were assumed to support the ordering and storage processes during sentence comprehension, respectively, were hypothesized in the reading of Chinese SRC and ORC sentences. If Chinese were a language that exhibits an ORC preference as predicted by the DLT, we would expect to observe greater activation in the LIFG and the LSTG during the comprehension of the more difficult Chinese SRC sentences than the easier ORC sentences. On the other hand, if the opposite activation pattern were observed to support an SRC preference in Chinese, the DLT would be deemed inadequate to account for the RC processing asymmetry in Chinese. In addition to examining the neural correlates underlying the comprehension of Chinese RC sentences, which has not been directly investigated before, in this study we also explored the effective connectivity between the LIFG and the LSTG by using a Granger Causality analysis, in order to enhance the understanding of how those brain areas process sentences in an interactive way.

2. Methods

2.1. Participants

Twenty-one healthy, right-handed native Chinese speakers were paid to attend the present study. Two subjects were excluded because of

low accuracy on sentence comprehension questions (71.88% and 68.75%), leaving 19 participants (9 females; aged from 19 to 29 years, mean age = 23 years, standard deviation = 2.3 years) whose data were further analyzed. All the participants had normal or corrected-to-normal vision and no history of neurological or psychiatric disorders. The experimental protocol for the study was approved by the Research Ethics Committee of National Taiwan University (NTU), Taipei, Taiwan. The study was conducted in accordance with the recommendations of the Social and Behavioral Research Ethical Principles and Regulations of NTU with signed written informed consent from all participants.

2.2. Materials and design

Sixty-four pairs of experimental sentences with Chinese SRCs and ORCs were created (thus, 128 sentences in total). The sentences were equal in overall word length across conditions, and each sentence was split into six frames (with each frame containing one to three characters), corresponding to the behavioral self-paced reading paradigm we employed before (Xu, Duann, Hung, & Wu, 2019). Furthermore, all of the experimental sentences had two animate nouns or noun phrases, which were equally likely to be the subject or object of the verb. In other words, all of the experimental sentences were semantically reversible (see Example A and B). A pilot study of plausibility rating with another group of 20 participants who did not attend this fMRI study showed no significant differences in the naturalness of these two types of sentences (t_1 (19) = 1, $p = 0.330$, t_2 (1 2 6) = 0.982, $p = 0.328$).

These 64 experimental sentence pairs with both Chinese SRCs and ORCs were evenly divided into two lists, each of which contained 32 SRC sentences and 32 ORC sentences, with the requirement that the SRC and ORC sentences in a pair were in different lists. Half of the participants received one list of the sentences whereas the other half of the participants received the other list. Thus, no participants encountered both the SRC and ORC sentences in a pair during the experiment. All of the 64 sentences in a list were then evenly divided into four sub-lists, one for each of the four fMRI runs. The order of the sentences in a sub-list was completely randomized across participants.

(A) The example of Chinese SRC sentences					
攻擊	張三	的	司機	違反了	規定
gōngjī	Zhāngsān	de	sījī	wěifǎnle	guīdìng
attack	Zhangsan	de	driver	violate	rule
'The driver who attacked Zhangsan has violated the rules.'					
(B) The example of Chinese ORC sentences					
張三	攻擊	的	司機	違反了	規定
Zhāngsān	gōngjī	de	sījī	wěifǎnle	guīdìng
Zhangsan	attack	de	driver	violate	rule
'The driver who Zhangsan attacked has violated the rules.'					

2.3. Procedure

A mixed-trial fMRI design was adopted in the present study. The main fMRI task was sentence comprehension, in which the stimuli were presented frame by frame at a steady rate to avoid possible eye movement during natural reading. The participants were asked to answer a probe question regarding the meaning of the preceding experimental sentence. The probe question was designed with the structure of 'who did what to whom' to test whether the participants comprehend the meaning of the sentence. Fig. 1A depicts the timeline of a single trial of the sentence comprehension task. Specifically, the time interval before the sentence onset was jittered between 2 s and 4 s. Each trial started with a fixation cross presented for 300 ms, followed by a blank screen for 100 ms. Six frames were then successively presented on the screen for 500 ms with 100 ms inter-stimulus intervals. Each Chinese character subtended a visual angle of approximately 1° vertically and was presented in DFKai-SB font in white against a black background. After the presentation of a whole target sentence, the participant made a true/false judgment in response to the probe question by pressing one of two buttons with the thumb of the left or the right hand within 4 s. The time interval between the target sentence and the probe question was also jittered between 2 s and 4 s. Among all the probe questions, half of the correct answers were 'true', while the other half of the correct answers were 'false'. Feedback regarding each participant's responses was given right after each response.

To identify the brain areas related to the comprehension of RC sentences more than to those related to the basic visual processing of stimuli, we used a visual orientation task as a baseline condition that was similar to the control task used in Constable et al. (2004). Specifically, visual stimuli containing two rows of five arrows with the same or different orientations (e.g., > > > > > and < < < < <), one immediately above the other, were presented. The participants were required to make the same/different orientation judgment by pressing the appropriate button using their left/right thumb. For this baseline condition, each trial began with a 1000-ms fixation, followed by the presentation of the two rows of arrows requiring the participant to make a judgment within 3 s.

The whole experiment consisted of four runs with an equal length (180 scans), and between runs participants could take a break. The timeline of one experimental run is shown in Fig. 1B. Each run contained six blocks, with two blocks of Chinese SRC reading (each block included four sentences), two blocks of Chinese ORC reading (each also for four sentences), and two blocks of visual orientation judgment (VO). In each run, the sentence comprehension and baseline conditions were presented in a pseudo-randomized order. Before the formal experiment, a practice session was conducted to ensure that the participants correctly followed the instructions for the tasks. The Cogent Matlab toolbox (http://www.vislab.ucl.ac.uk/cogent_2000.php) was used to deliver the stimuli in the experiment.

2.4. Imaging protocol

The MRI images were acquired using a 3T scanner (MAGNETOM Skyra, Siemens, Erlangen, Germany) with a 64-channel whole-head coil located at National Cheng-Chi University, Taipei, Taiwan. The participants' heads were immobilized with a vacuum-beam pad in the scanner. Functional, blood oxygenation level-dependent (BOLD) signals were acquired with a T2*-weighted echo planar imaging (EPI) sequence with the following parameters: slice thickness of 3.4 mm and no gap, in-plane resolution of 3.4375 × 3.4375 mm, and TR/TE/flip angle = 2000 ms/70 ms/77°. The field-of-view was 220 × 220 mm, and the acquisition matrix was 64 × 64. Thirty-four oblique-axial slices paralleled to the anterior commissure-posterior commissure (AC-PC) line were acquired to cover the whole brain. The first five volumes of each functional run were discarded for signal equilibrium, and a total of one hundred and eighty image volumes were acquired in each of the four runs. To obtain fine-grained localization information of the fMRI activity, a high-resolution anatomical brain image of each participant was obtained using a T1-weighted sequence (TR = 2530 ms/TE = 3.3 ms/flip angle = 7°, bandwidth = 200 Hz/pixel). This sequence used 192 sagittal slices to cover the whole brain and resulted in an isotropic spatial resolution of $1 \times 1 \times 1\ mm^3$.

2.5. Imaging data analysis

The fMRI data were processed with the Statistical Parametric Mapping package (SPM8) developed by the Wellcome Trust Center for Neuroimaging at University College London. The image data with error responses were excluded from the analysis. Images of each participant were first adjusted for slice timing so that each slice's time series was temporally aligned after correcting the shifts between the slice acquisitions in a volume. Then, the image volume series was realigned to the middle image volume of each run to correct for head movements

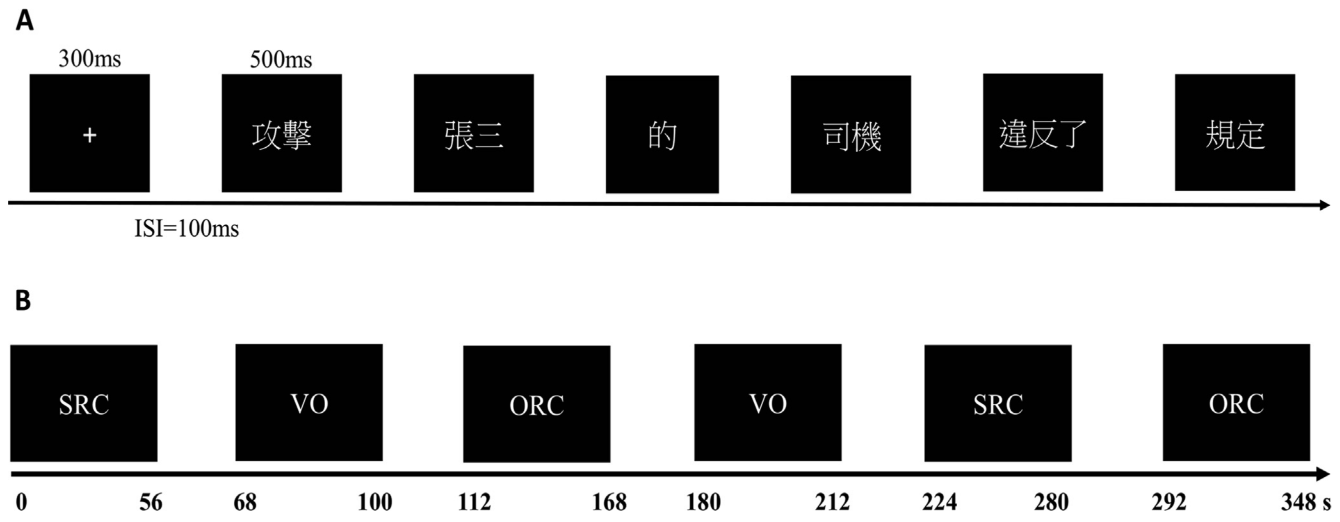

Fig. 1. Experimental procedure. (A) The timeline of one sentence trial. A fixation cross was shown on the screen, followed by the presentation of each frame of the whole sentence. Afterwards, participants had to press a button to make true/false judgments based on the meaning of the previous target sentence. Finally, visual feedback was presented. (B) The timeline of one run in the experiment. Each run contained six blocks, with two blocks of each type of sentences (SRC or ORC) and baseline (VO) conditions. The block order of three conditions in each run was pseudo-randomized. Note: VO = visual orientation; SRC = subject-extracted relative clause; ORC = object-extracted relative clause.

(Andersson, Hutton, Ashburner, Turner, & Friston, 2001; Hutton et al., 2002). A mean functional image volume was created for each participant and each run from the realigned image volumes and spatially normalized to an MNI (Montreal Neurological Institute) EPI template using a nonlinear affine transformation (Ashburner & Friston, 1999; Friston, Holmes, et al., 1995). The normalization parameters determined from the mean functional volume were then applied to the corresponding functional image volumes for each participant. Finally, the images were smoothed with a Gaussian kernel of 8 mm full-width at half-maximum.

An analytical, statistical design was constructed for each participant, using the general linear model (GLM) with the parameters consisting of the onsets of the target sentences and visual orientation, convolved with a canonical hemodynamic response function and its temporal derivative (Duann, Ide, Luo, & Li, 2009; Friston, Ashburner, et al., 1995). The six-degree-of-freedom realignment parameters were also included in the model. The data was high-pass filtered temporally (128 s cutoff) to remove low-frequency signal drifts. In the first-level analysis, we constructed, for each participant, two contrasts between each of the sentence comprehension conditions against the visual orientation (VO) task (i.e., SRC vs. VO and ORC vs. VO). The 'con' or contrast images of the first-level analysis were then utilized for the second-level group statistics using a one-sample T-test. Considering that Bonferroni correction is too conservative for most of the fMRI studies with potentially subtle BOLD activations, here we adopted the family-wise error rate (FWER) for correcting the multiple comparison errors. The FWER was achieved by adjusting the significance level according to the formula, $1\text{-}(1\text{-}\alpha_{IT})^c$, where α_{IT} is the alpha level for an individual test (e.g., 0.05) and c is the number of comparisons. Therefore, the brain activation at the single-subject level was thresholded at $p < 0.05$, corrected for multiple comparisons using FWER (Friston, Ashburner, et al., 1995) and was visualized using the xjView toolbox (http://www.alivelearn.net/xjview) to identify the target brain areas at least with the cluster size > 10 voxels. We then used MarsBaR (http://marsbar.sourceforge.net/) to extract the effect size of activity changes in identified brain regions for each participant (Brett, Johnsrude, & Owen, 2002).

2.6. Granger causality analysis

In addition to the observation of the activation pattern resulting from a regression model, temporal precedence information can also be explored by computing Granger Causality (GC) that identifies the active brain areas that may play as sources or targets of the directed influences in relation with other brain areas from fMRI data (Granger, 1969). This method has been widely used to estimate 'causal' influence between sets of fMRI time series data (Deshpande, LaConte, James, & Hu, 2009; Ding, Bressler, Yang, & Liang, 2000; Duann et al., 2009; Goebela, Roebroecka, Kimb, & Formisano, 2003; Sato et al., 2009). Technically, the directional influence between two different brain areas can be estimated using the GC based on the delayed version of the temporal information of the two brain areas. For example, there are two time courses from two different brain areas, x[n] and y[n]. We first compute the variance, ε, of the time series data y[n] being accounted for by the past values of y[n] using an autoregressive (AR) model. Then, we further additionally incorporate the past values of the other time series data x[n] to account for the signal y[n] and obtain the residual variance ε'. If the residual variance ε' is significantly smaller than the residual variance ε, the signal x[n] is called to Granger-cause the signal y[n] by the definition of the GC (Roebroeck, Formisano, & Goebel, 2005). Using the same approach, we can further compute the residual variance of the x[n] using an AR model with and without the involvement of the past values of the signal y[n] to determine if y[n] significantly Granger-causes the signal x[n]. Because the result that x[n] Granger-causes y[n] does not imply the relationship of y[n] Granger causing x[n], the causality estimation is thus directional and called effective connectivity. As introduced, the GC is mainly computing the dependence of any of two time-series data with delay in time to estimate the temporal dependence and the directional information flow between two nodes, which is different from the GLM that has been used to compute the multi-regression model of the fMRI time series data at each of the single voxel against a combination of reference functions, such as the BOLD reference time course obtained by convolving the experimental paradigm with the canonical hemodynamic response function, six degree-of-freedom movement estimation, etc., in the design matrix. Therefore, in this study, after the activation analysis using the GLM model to identify the brain regions that were significantly involved in sentence processing, we further used those results to inform effective connectivity analyses that allow us to investigate task-specific changes in the effectivity connectivity between the LIFG and the LSTG by means of a GC analysis.

Specifically, the conditional (SRC and ORC) time courses of

significantly activated brain areas were further subjected to the GC analysis. To extract the conditional time courses, we used the MarsBaR in SPM8 with the regions of interest (ROIs) of 5-mm radius applied to the peak voxels of significantly activated brain areas (i.e., LIFG and LSTG) to find the average time courses. Then, the time courses were segmented according to the time periods of the SRC and ORC conditions to obtain the conditional time courses for computing the GC between the brain areas under each of the two conditions. The GC was computed using the bivariate AR model on the time courses from each pair of the activated brain areas (i.e., LIFG and LSTG). First, we computed the GC between those two ROIs by concatenating the time courses from all four runs together and extracting from each of the two sentence comprehension conditions. Then, we calculated the residual variance using the bivariate AR model with the optimal lag number determined for each time course separately. The F values used to compare the residual variance with and without the other time courses being included in the bivariate AR model, were computed for the time course of each pair of the activated brain areas, and the direction of the connectivity between areas was further determined at the significance level of $p < 0.01$ for each participant. Finally, the binomial test with a significance level of $p < 0.05$ was used to summarize the group results regarding the direction of the connectivity between the LIFG and LSTG. To validate the observed connectivity between the LIFG and the LSTG, we further examined the significance of the difference between the GC results, namely, $F_{LIFG \to LSTG}$ versus $F_{LSTG \to LIFG}$, using a Wilcoxon Signed Ranks test (Roebroeck et al., 2005).

3. Results

3.1. Behavioral results

The accuracy and reaction time (RT) of the responses to comprehension questions were recorded. Only the RT of accurate responses and after removal of the outliers beyond two standard deviations (SD) around the mean was included for further analysis. Generally, the comprehension accuracy (mean ± SD) for all the sentences across participants was 94.33 ± 3.90%, with an accuracy of 94.57 ± 3.10% for the SRC sentences and an accuracy of 94.08 ± 4.65% for the ORC sentences. No significant difference was found between these two conditions (t (18) = 0.389, p = 0.702). With regard to RT (mean ± SD), there was also no significant difference (t (18) = −0.060, p = 0.953) between the SRC (1137.88 ± 194.55 ms) and the ORC (1139.17 ± 161.62 ms) sentences.

3.2. Imaging results

3.2.1. Whole-brain analysis

Table 1 summarizes the coordinates of peak activation in the regions that are activated at the significance level of $p < 0.05$ (FWER corrected) for each type of sentences compared to the visual baseline. The activation pattern associated with each type of sentence contrasted with that associated with the visual baseline is illustrated in Fig. 2. As shown, both SRC and ORC sentences recruited similar brain areas for sentence comprehension, and only a left-lateralized frontotemporal neural network was identified.

After contrasting each type of sentences with the visual baseline, we further conducted a direct contrast analysis between the two conditions, namely, (SRC - VO) vs. (ORC - VO), to highlight brain activation associated with sentence comprehension. As shown in Table 2, greater activation in the LIFG and the LSTG was significantly evoked by the SRC sentences than by the ORC sentences at the threshold of $p < 0.001$ (Fig. 3, with the coordinates of the peak activation in these regions). On the other hand, no brain areas showed higher activation in the ORC condition than the SRC condition, as the contrast of (ORC - VO) vs. (SRC - VO) did not reveal any significant results.

3.2.2. Granger causality (GC) results

As shown in Fig. 3, there were two regions significantly activated by the contrast between (SRC - VO) and (ORC - VO). The peak activations were located in the brain areas of the LIFG (x = −34, y = 28, z = −4) and the LSTG (x = −60, y = −50, z = 16). Because only two significantly activated brain areas were found in the contrast analysis, a bivariate AR model was used to conduct the GC analysis. The results of the GC analysis indicated that during the processing of the SRC sentences, which were more difficult to process than the ORC sentences according to the whole-brain analysis, the LIFG significantly Granger-caused the LSTG at the group level using a binomial test (p = 0.0318), while the GC in the same direction when reading ORC sentences was not significant (p = 0.9682), as shown in Fig. 4. On the other hand, the GC from the LSTG to LIFG was far from significance in both conditions (SRC: p = 0.6762; ORC: p = 0.6762), nor was the direct comparison of the GCs between the conditions significant (Z = 1.288, p = 0.210). Moreover, the Wilcoxon Signed Ranks test further confirmed that during the process of the SRC condition, the strength of the connectivity from the LIFG to the LSTG was significantly stronger than that from the opposite direction (Z = 2.334, p = 0.018). Besides, the strength of the connectivity from the LIFG to the LSTG in the SRC condition was also significantly stronger than that in the ORC condition (Z = 2.575, p = 0.008).

Table 1
Locus and extent of peak activation in brain regions during the comprehension of the SRC and ORC sentences.

Scan	Anatomical Region	BA	Voxels	MNI coordinate (mm) x	y	z	Z-value	Sidedness
SRC - VO	Superior temporal gyrus	22	147	−54	−42	2	6.55	L
	Superior temporal gyrus	22	50	−50	−54	12	5.45	L
	Temporal pole	38	10	−50	6	−14	5.43	L
	precentral gyrus	6	25	−40	−2	44	5.38	L
	Fusiform Gyrus	37	75	−40	−56	−22	5.34	L
	Inferior frontal gyrus	47	12	−36	28	−6	5.02	L
ORC - VO	Superior temporal gyrus	22	120	−54	−40	2	6.04	L
	precentral gyrus	6	23	−40	−4	44	5.40	L
	Middle temporal gyrus	21	27	−56	0	−16	5.25	L
	Fusiform Gyrus	37	28	−40	−58	−20	5.07	L
	Posterior cingulate cortex	23	12	−2	−58	18	5.02	L

The contrasts are significant at family-wise error rate (FWER) threshold of $p < 0.05$, cluster size > 10 voxels. Coordinates are reported in MNI space and refer to the peak Z scores for each region. SRC = subject-extracted relative clause; ORC = object-extracted relative clause; VO = visual orientation; BA = brodmann area; L = left.

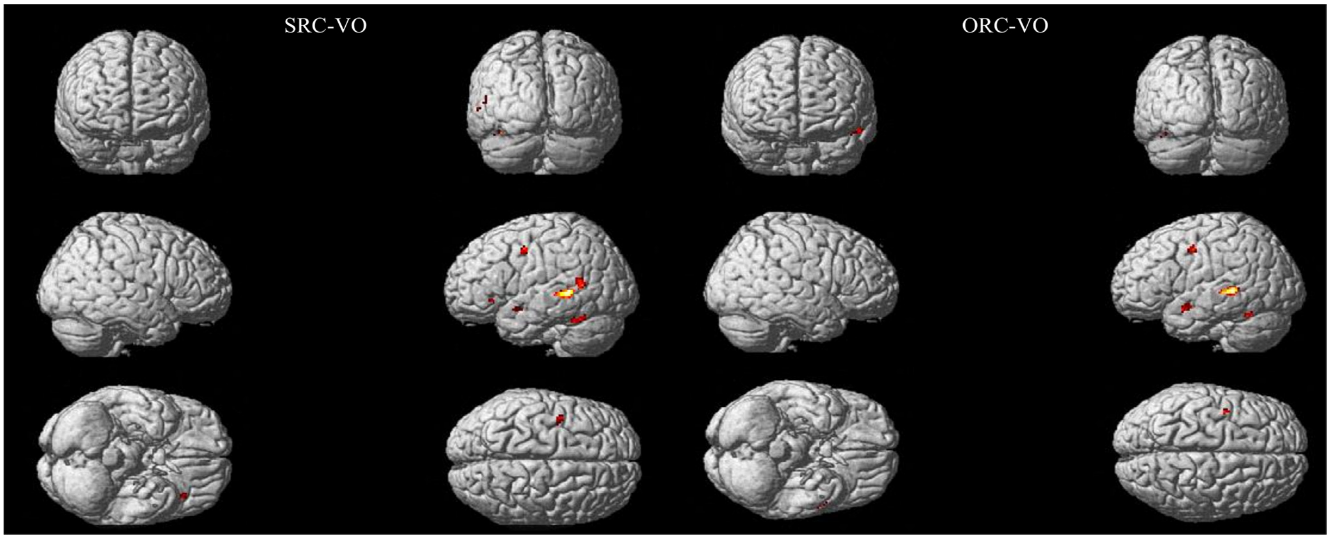

Fig. 2. Volume renderings of the (SRC - VO) and (ORC - VO) effect, respectively ($p < 0.05$, FWER corrected, cluster size > 10 voxels). The results show that a left-lateralized neural network is both identified in these two contrasts. Note: VO = visual orientation; SRC = subject-extracted relative clause; ORC = object-extracted relative clause; FWER = family-wise error rate.

Table 2
Locus and extent of peak activation in brain regions under the comparison between (SRC - VO) and (ORC - VO).

Scan	Anatomical region	BA	Voxels	MNI coordinate (mm) x	y	z	Z-value	Sidedness
(SRC - VO)	IFG	47	139	−34	28	−4	4.34	L
vs.	STG	22	51	−60	−50	16	3.56	L
(ORC - VO)								

The contrast was significant at the threshold of $p < 0.001$, cluster size > 30 voxels. Coordinates are reported in MNI space and refer to the peak Z scores for each region. SRC = subject-extracted relative clause; ORC = object-extracted relative clause; VO = visual orientation; IFG = inferior frontal gyrus; STG = superior temporal gyrus; BA = brodmann area; L = left.

4. Discussion

This study presents neuroimaging evidence, for the first time in the literature, to reveal the neural correlates underlying the processing of Chinese RC sentences and to provide clear support for an ORC preference in Chinese, particularly in the mechanisms subserved by the LIFG and the LSTG. Contrary to the common observations of an SRC preference in other languages, our results demonstrated that Chinese SRC sentences were more difficult to comprehend than ORC sentences, as reflected in the increased activation of the LIFG and the LSTG indicated by the whole-brain analysis. Furthermore, significant effective connectivity between the LIFG and the LSTG was only found during the comprehension of Chinese SRC rather than ORC sentences. These results are interpreted as showing a greater processing load in the storage and the ordering processes during the comprehension of Chinese SRC than ORC sentences, which are in line with the prediction of the Dependency Locality Theory (DLT) that postulates contribution from working memory to sentence comprehension.

Chinese RC sentences do not have the on-site case-role specification of the referential noun phrases. As a result, multiple referents need to be stored before the integration of multiple phrases and argument-verb relations. Various demands in the ordering and storage processes induce different processing difficulty during the comprehension of Chinese SRC and ORC sentences. That is, when comprehending Chinese SRC sentences, a greater processing load is needed to maintain the predicted syntactic head and to reorder the words to fill in the subject gap until the completion of successful comprehension. On the other hand, the reading of Chinese ORC sentences only requires the incremental combination of the upcoming words across a short filler-gap distance. It has been argued that the preference for the ORC should be observed in Chinese according to the DLT. Supporting findings from neuroimaging research for the assumptions of the DLT have been provided in other languages (e.g., Ben-Shachar et al., 2003; Caplan et al., 2008; Constable et al., 2004; Friederici, 2011; Friederici et al., 2009; Kemmerer, 2012; Kuhnke et al., 2017; Meyer et al., 2012; Meyer, Grigutsch, Schmuck, Gaston, & Friederici, 2015; Nishitani, Schürmann, Amunts, & Hari, 2005), but the neuroimaging evidence from Chinese has not been reported until the present study.

The present results clearly illustrated that varying demands in the processing of different sentences could modulate task-specific neural activity in the left-hemispheric frontotemporal regions as demonstrated before (e.g., Friederici, 2011; Santi & Grodzinsky, 2010; Skeide et al., 2016). Specifically, stronger brain activation was significantly elicited by Chinese SRC than ORC sentences in the LSTG, which might help store information, particularly the meaning of words or other 'relational' information, during sentence comprehension (e.g., Bornkessel et al., 2005; Friederici, 2011; Grodzinsky & Friederici, 2006). In the meantime, increased activation in the LIFG that might play a role in syntactic realization of hierarchical dependencies by ordering the arguments (e.g., Meyer et al., 2012; Kuhnke et al., 2017) was also found in response to Chinese SRC sentences with non-canonical word order in the RC compared to Chinese ORC sentences with canonical word order in the RC. These findings agree with previous neuroimaging work that emphasizes the importance of the LIFG and the LSTG for RC sentence processing (e.g., Bornkessel-Schlesewsky & Schlesewsky, 2013; Friederici, 2011; Hagoort, 2014; Meyer et al., 2012; Pattamadilok, Dehaene, & Pallier, 2016), highlighting their roles in the ordering and storage processes, respectively.

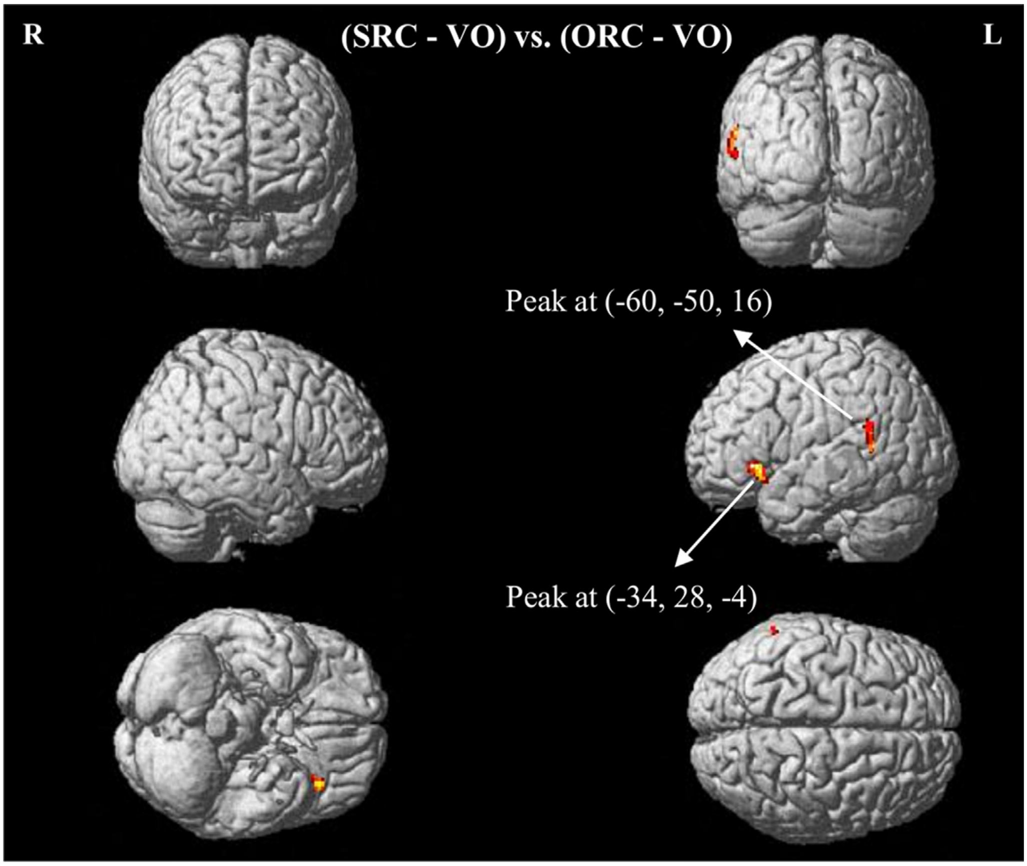

Fig. 3. Volume rendering of the (SRC - VO) vs. (ORC - VO) effect ($p < 0.001$, uncorrected, cluster size > 30 voxels). The result indicates that greater activation in the left inferior frontal gyrus (LIFG) and the left superior temporal gyrus (LSTG) is significantly evoked by SRC sentences in comparison to ORC sentences. Note: VO = visual orientation; SRC = subject-extracted relative clause; ORC = object-extracted relative clause.

In addition to the conventional SPM approach, we also employed GC to investigate the functional connections between regions of the cortical networks that mediate sentence comprehension. We found evidence showing a direct influence of the LIFG on the LSTG during the comprehension of the more difficult SRC sentences rather than the ORC sentences. Specifically, it was observed that the effective connectivity from the LIFG to the LSTG, but not for the opposite direction, significantly facilitated the reading of the SRC sentences. In contrast, the same connectivity between the LIFG and the LSTG was not significant in either direction during the comprehension of the ORC sentences. Previous studies have demonstrated that enhanced effective connectivity between the LIFG and the LSTG is associated with increased efficiency of sentences processing (e.g., Papoutsi, Stamatakis, Griffiths, Marslen-Wilson, & Tyler, 2011). Moreover, studies on visual

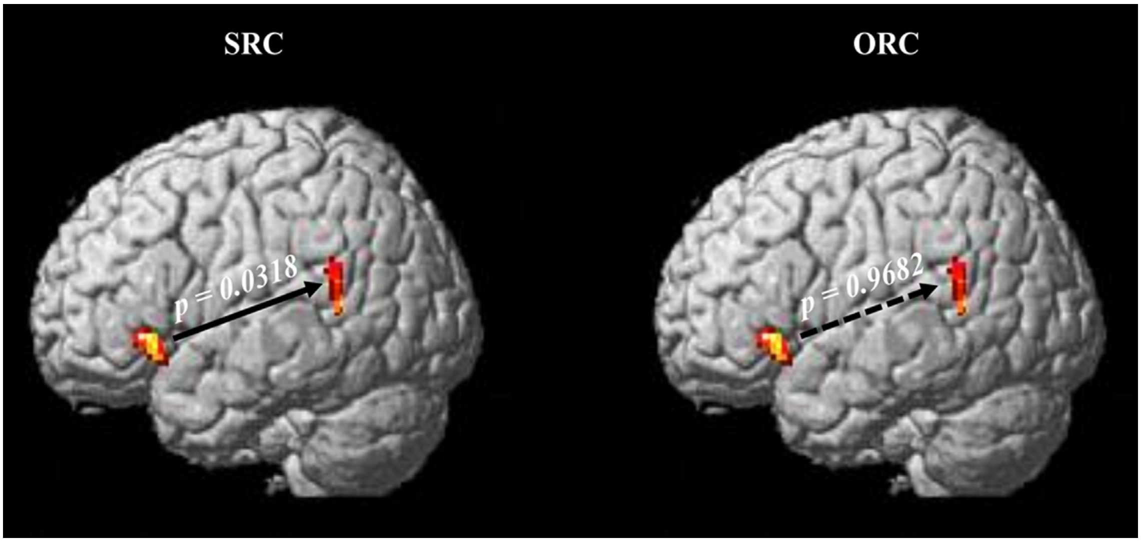

Fig. 4. Effective connectivity between the LIFG and the LSTG in SRC and ORC conditions. The results show that during the reading of Chinese SRC sentences, the LIFG significantly Granger-causes the LSTG ($p = 0.0318$), while the GC in the same direction when reading ORC sentences is not significant ($p = 0.9682$). These findings suggest that enhanced connectivity between the LIFG and the LSTG prominently facilitates the comprehension of more difficult SRC sentences rather than the ORC sentences. Note: LIFG = left inferior frontal gyrus; LSTG = left superior temporal gyrus; SRC = subject-extracted relative clause; ORC = object-extracted relative clause.

and verbal memory have shown that sustained activation of representations in the LSTG is under frontal top-down control (e.g., Fiebach, Rissman, & D' Esposito, 2006; Sreenivasan, Curtis, & D' Esposito, M. , 2014). Similarly, the causal top-down influence of the frontal cortex on the temporal cortex has also been found in a semantic priming paradigm (Hartwigsen et al., 2017) and a speech perception task (Cope et al., 2017). Therefore, based on the previous findings as well as the present results, we propose that the effective connectivity from the LIFG to the LSTG may play an important role in the comprehension of complex sentences. Further evidence is still needed to provide support for this claim.

It should be noted that there are conflicting and inconclusive results about Chinese RC preference in the literature, possibly due to the multiple factors involved in sentence comprehension, such as thematic orders (e.g., Lin, 2014), discourse context (e.g., Yang & Perfetti, 2006, Gibson & Wu, 2013), and animacy (e.g., Wu, Kaiser, & Andersen, 2012; He & Chen, 2013). In the present study, to determine the contribution of the syntactic structure to RC processing while minimizing the influence from other potential factors, we focused on the processing of the basic form of Chinese RC with animated nouns, equal sentence length, and identical words in different orders. The findings are generally in line with the memory-based account (i.e., the DLT), which supports that when considering memory loads in the ordering and storage processes for the comprehension of RC sentences with different syntactic structures, Chinese ORC is easier and required less processing resources than Chinese SRC. However, such processing preference might only apply to the incremental build-up processing of syntactic structure.

Besides, it has been reported that for the Chinese SRC construction ('Verb + NP1 + de + NP2′), there is a temporary ambiguity between a complement clause (CC) analysis and a head-final RC analysis (see the details from Hsieh, Boland, Zhang, and Yan (2009) and Zhang, Zhang, and Hua (2000)). On the other hand, the involvement of the LIFG and the LMTG (i.e., left middle temporal gyrus), which are much overlapped with the brain areas identified in the present study, have been frequently observed in research of ambiguity resolution (e.g., Acheson & Hagoort, 2013; Mason, Just, Keller, & Carpenter, 2003; Rodd, Longe, Randall, & Tyler, 2010; Zempeni, Renken, Hoeks, Hoogduin, & Stowe, 2007). Thus, from the present experimental protocol, we might not fully rule out the possibility that increased activation in these areas is partially due to the detection of temporal ambiguity (if any) involved in the SRC condition. Nevertheless, based on previous real-time eye-tracking or EEG studies on this issue, we deem that temporal ambiguity resolution is less likely to be the main reason for the present findings based on the following reasons.

First, it has been found that there is a strong tendency to initially interpret the Chinese 'verb + NP1 + de + NP2′ construction toward an RC instead of a CC structure (e.g., Hsieh et al., 2009; Lin & Garnsey, 2011; Pu, 2007; Zhang et al., 2000). It is also demonstrated that resolving ambiguity for the 'verb + NP1 + de + NP2′ construction was costly only when the disambiguation was toward the more complex, less preferred CC structure, rather than toward the more preferred RC structure (e.g., Hsieh et al., 2009; Zhang et al., 2000). With the eye-tracking technique, Hsieh et al. (2009) investigated the temporal ambiguity of 'verb + NP1 + de + NP2′ construction and found that when NP1 and NP2 were both animate, the reader tended to initially interpret the sentence as the RC structure. On the other hand, the additional computation, as reflected in significantly increased eye-movement measures, was required for eventually disambiguating the sentence as the less preferred CC structure. Based on these previous findings, in the present fMRI study we only adopted SRC and ORC sentences while there was no CC structure in the experimental stimuli. In addition, all the target RC sentences employed both animate NP1 and NP2, to the greatest extent facilitating the RC reading.

Second, the eye-tracking or EEG technique with a high temporal resolution has also been adopted to investigate the processing difference between Chinese SRCs and ORCs. The corresponding results are found to be more compatible with the interpretation of processing difference in filler-gap dependency rather than temporal ambiguity resolution. According to the DLT, the processing difference between SRCs and ORCs is mainly due to varying memory loads required for the understanding of filler-gap dependency, thus greater processing cost is predicted in the relative marker 'de' and/or head noun regions of SRCs (Gibson, 1998). The evidence from the eye-tracking study has shown that the gaze duration, as well as the regression-path duration, for head noun regions (i.e., the NP2 position) in Chinese ORC sentences was shorter than that in SRC sentences, indicating that Chinese ORC sentences were easier to process than SRC sentences (Sung et al., 2016). Packard, Ye, and Zhou (2010) carried out an ERP study to explore the temporal dynamics of the comprehension on Chinese SRC and ORC sentences and also found a significantly greater positivity for SRCs over ORCs in the P600 component of the relative marker 'de', due to a higher filler-gap integration required in Chinese SRC sentences. These results, as well as those from the present study employing similar stimuli, are thus consistent with the predictions of the DLT.

Last but not least, in the literature many studies attempting to compare Chinese SRCs with ORCs have provided valuable findings to increase the understandings of how structural complexity affects sentence comprehension (*Behavioral studies*: Chan, Matthews, & Yip, 2011; Chen et al., 2008; Gibson & Wu, 2013; Vasishth et al., 2013; Hsiao & Gibson, 2003; Hsu, Hermon, & Zukowski, 2009; Lin, 2014; *Neurophysiological studies*: Bulut, Cheng, Xu, Hung, & Wu, 2018; Packard et al., 2010; Yang & Perfetti, 2006; Yang, Perfetti, & Liu, 2010). Due to a lack of relevant neuroimaging studies in this field, we thus conducted an fMRI study to reveal how comprehension of sentences with different structural complexity was implemented in the human brain. Neuroimaging findings from other languages have demonstrated that involvement of the LIFG and the LSTG is critical to processing the sentence with embedded structure (e.g., Caplan et al., 2008; Constable et al., 2004; Cooke et al., 2002; Friederici et al., 2006; Friederici, 2011; Just et al., 1996; Santi & Grodzinsky, 2010; Skeide et al., 2016), which are consistent and also helpful to understand the associated functions of the similar activation pattern identified in the present study. Along this line, we further reason that the observed processing difficulty in the SRC condition may mainly reflect the processing demands of syntactic computations that link a verb to its dislocated argument through a trace in the present and previous studies (e.g., Bulut et al., 2018; Chen et al., 2008; Gibson & Wu, 2013; Hsiao & Gibson, 2003; Packard et al., 2010; Yang & Perfetti, 2006; Yang et al., 2010), rather than the disambiguation process between the RC and CC readings. Future work adopting the state-of-the-art techniques, e.g., the simultaneous fMRI-eye tracking or fMRI-EEG measures, may help differentiate aspects of sentence processing residing in a similar network.

In the present study, the involvement of the LIFG but without the classic Broca's area is identified in sentence processing. It has been argued that the LIFG can be classified into three functional parts, namely, the pars opercularis (BA44), the anterior inferior pars triangularis (BA 45) and the pars orbitalis (BA 47) (e.g., Christensen, Kizach, & Nyvad, 2013). Traditionally, BA 44 and BA 45 were regarded as the core areas associated with sentence processing (Constable et al., 2004; Friederici, Meyer, & Cramon, 2000; Hagoort et al., 1999; Pugh et al., 1996). However, some studies have reported increased activity in BA 44/45 extending to BA 47 when processing complex sentences with a long filler-gap distance (Burholt Kristensen, Engberg-Pedersen, Højlund Nielsen, & Wallentin, 2013; Cooke et al., 2002). In addition, lesion research has also found that the dysfunction of BA 47 causes difficulty in comprehending hierarchically complex sentences (Dronkers, Wilkins, Van Valin, Redfern, & Jaeger, 2004). Taken together, the previous and present findings suggest that besides the classic Broca's area, BA 47 might also be involved in sentence processing. Further research is still needed to elucidate the specific functions of distinct sub-regions of the LIFG, as well as the interactions of these brain areas in processing hierarchically embedded sentences.

In sum, the present findings reveal the critical roles of the LIFG and the LSTG during the processing of Chinese RC sentences and provide clear neuroimaging evidence in support of an ORC preference in Chinese. Moreover, enhanced effective connectivity from the LIFG to the LSTG prominently contributes to the comprehension of Chinese SRC sentences that are more complex than Chinese ORC sentences. Therefore, besides a particular activation pattern in language-related regions, the extensive collaboration between those regions may also play an important role in sentence comprehension.

Acknowledgments

This research was supported in part by the grants from the Ministry of Science and Technology (MOST 102-2628-H-008-002-MY3; MOST 106-2410-H-008-054; MOST 107-2221-E-008-032-MY2), Taiwan.

Declaration of Competing Interest

The authors declare that the research was conducted in the absence of any commercial or financial relationships that could be construed as a potential conflict of interest.

References

Acheson, D. J., & Haggort, P. (2013). Stimulating the brain's language network: Syntactic ambiguity resolution after TMS to the inferior frontal gyrus and middle temporal gyrus. *Journal of Cognitive Neuroscience, 25*(10), 1664–1677. https://doi.org/10.1162/jocn_a_00430.

Andersson, J. L. R., Hutton, C., Ashburner, J., Turner, R., & Friston, K. (2001). Modeling geometric deformations in EPI time series. *NeuroImage, 13*(5), 903–919. https://doi.org/10.1006/nimg.2001.0746.

Ashburner, J., & Friston, K. J. (1999). Nonlinear spatial normalization using basis functions. *Human Brain Mapping, 7*(4), 254–266. https://doi.org/10.1002/(SICI)1097-0193(1999) 7:4 < 254::AID-HBM4 > 3.0.CO;2-G.

Ben-Shachar, M., Hendler, T., Kahn, I., Ben-Bashat, D., & Grodzinsky, Y. (2003). The neural reality of syntactic transformations: Evidence from functional magnetic resonance imaging. *Psychological science, 14*(5), 433–440. https://doi.org/10.1111/1467-9280.01459.

Ben-Shachar, M., Palti, D., & Grodzinsky, Y. (2004). Neural correlates of syntactic movement: Converging evidence from two fMRI experiments. *NeuroImage, 21*(4), 1320–1336. https://doi.org/10.1016/j.neuroimage.2003.11.027.

Bornkessel, I., Zysset, S., Friederici, A. D., Cramon, D. Y. V., & Schlesewsky, M. (2005). Who did what to whom? The neural basis of argument hierarchies during language comprehension. *NeuroImage, 26*(1), 221–233. https://doi.org/10.1016/j.neuroimage.2005.01.032.

Bornkessel-Schlesewsky, I., & Schlesewsky, M. (2013). Reconciling time, space and function: A new dorsal-ventral stream model of sentece comprehension. *Brain and Language, 125*(1), 60–76. https://doi.org/10.1016/j.bandl.2013.01.010.

Brett, M., Johnsrude, I. S., & Owen, A. M. (2002). The problem of functional localization in the human brain. *Nature, 3*(3), 243–250. https://doi.org/10.1038/nrn756.

Bulut, T., Cheng, S.-K., Xu, K., Hung, D. L., & Wu, D. H. (2018). Is there a processing preference for object relative clauses in Chinese? Evidence from ERPs. *Frontiers in Psychology, 1–18*. https://doi.org/10.3389/fpsyg.

Burholt Kristensen, L., Engberg-Pedersen, E., Højlund Nielsen, A., & Wallentin, M. (2013). The influence of context on word order processing – An fMRI study. *Journal of Neurolinguistics, 26*(1), 73–88. https://doi.org/10.1016/j.jneuroling.2012.05.001.

Caplan, D., Stanczak, L., & Waters, G. (2008). Syntactic and thematic constraint effects on blood oxygenation level dependent signal correlates of comprehension of relative clauses. *Journal of Cognitive Neuroscience, 20*(4), 643–656. https://doi.org/10.1162/jocn.2008.20044.

Caplan, D., & Waters, G. S. (1999). Verbal working memory and sentence comprehension. *Behavioral Brain Science, 22*, 77–94.

Carreiras, M., Duñabeitia, J. A., Vergara, M., Cruz-Pavía, I.d.l., & Laka, I. (2010). Subject relative clauses are not universally easier to process: Evidence from Basque. *Cognition, 115*(1), 79–92. https://doi.org/10.1016/j.cognition.2009.11.012.

Chan, A., Matthews, S., & Yip, V. (2011). The acquisition of relative clauses in Cantonese and Mandarin. In Evan Kidd (Ed.). *The Acquisition of Relative Clauses: Processing, typology and function* (pp. 197–226). Amsterdam: John Benjamins Publishing Company.

Chen, B., Ning, A., Bi, H., & Dunlap, S. (2008). Chinese subject-relative clauses are more difficult to process than the object-relative clauses. *Acta Psychologica (Amst), 129*(1), 61–65. https://doi.org/10.1016/j.actpsy.2008.04.005.

Christensen, K. R., Kizach, J., & Nyvad, A. M. (2013). The processing of syntactic islands – An fMRI study. *Journal of Neurolinguistics, 26*(2), 239–251. https://doi.org/10.1016/j.jneuroling.2012.08.002.

Clahsen, H., & Featherston, S. (1999). Antecedent Priming at Trace Positions: Evidence from German Scrambling. *Journal of Psycholinguistic Research, 28*, 415–437. https://doi.org/10.1007/s10936-006-9041-8.

Constable, R. T., Pugh, K. R., Berroya, E., Mencl, W. E., Westerveld, M., Ni, W., et al. (2004). Sentence complexity and input modality effects in sentence comprehension: An fMRI study. *NeuroImage, 22*(1), 11–21. https://doi.org/10.1016/j.neuroimage.2004.01.001.

Cooke, A., Zurif, E. B., DeVita, C., Alsop, D., Koenig, P., Detre, J., et al. (2002). Neural basis for sentence comprehension: Grammatical and short-term memory components. *Human Brain Mapping, 15*(2), 80–94. https://doi.org/10.1002/hbm.10006.

Cope, T. E., Sohoglu, E., Sedley, W., Patterson, K., Jone, P. S., Wiggins, J., et al. (2017). Evidence for causal top-down frontal contributions to predictive processes in speech perception. *Nature Communications, 8*(1), 2154. https://doi.org/10.1038/s41467-017-01958-7.

Deshpande, G., LaConte, S., James, G. A., & Hu, S. P. (2009). Multivariate granger causality analysis of fMRI data. *Human Brain Mapping, 30*(4), 1361–1373. https://doi.org/10.1002/hbm.20606.

Ding, M., Bressler, S. L., Yang, W., & Liang, H. (2000). Short-window spectral analysis of cortical event-related potentials by adaptive multivariate autoregressive modeling: Data preprocessing, model validation, and variability assessment. *Biological Cybernetics, 83*(1), 35–45. https://doi.org/10.1007/s004229900137.

Dronkers, N. F., Wilkins, D. P., Van Valin, R. D., Jr., Redfern, B. B., & Jaeger, J. J. (2004). Lesion analysis of the brain areas involved in language comprehension. *Cognition, 92*(1–2), 145–177. https://doi.org/10.1016/j.cognition.2003.11.002.

Duann, J.-R., Ide, J. S., Luo, X., & Li, C.-S. R. (2009). Functional connectivity delineates distinct roles of the inferior frontal cortex and presupplementary motor area in stop signal inhibition. *Journal of Neuroscience, 29*(32), 10171–10179. https://doi.org/10.1523/JNEUROSCI.1300-09.2009.

Fiebach, C. J., Rissman, J., & D' Esposito, M. (2006). Modulation of inferotemporal cortex activation during verbal working memory maintenance. *Neuron, 51*(2), 251–261. https://doi.org/10.1016/j.neuron.2006.06.007.

Fiebach, C. J., Schlesewsky, M., & Friederici, A. D. (2001). Syntactic working memory and the establishment of filler-gap dependencies: Insights from ERPs and fMRI. *Psycholinguistic Research, 30*(3), 321–338. https://doi.org/10.1023/A:1010447102554.

Friederici, A. D. (2011). The brain basis of language processing: From structure to function. *Physiological Reviews, 91*(4), 1357–1392. https://doi.org/10.1152/physrev.00006.2011.

Friederici, A. D., Bahlmann, J., Heim, S., Schubotz, R. I., & Anwander, A. (2006). The brain differentiates human and non-human grammars: Functional localization and structural connectivity. *Proceedings of the National Academy of Sciences, 103*(7), 2458–2463. https://doi.org/10.1073/pnas.0509389103.

Friederici, A. D., Makuuchi, M., & Bahlmann, J. (2009). The role of the posterior superior temporal cortex in sentence comprehension. *Neuroreport, 20*(6), 563–568. https://doi.org/10.1097/WNR.0b013e3283297dee.

Friederici, A. D., Meyer, M., & Cramon, D. Y.v. (2000). Auditory language comprehension: An event-related fMRI study on the processing of syntactic and lexical information. *Brain and Language, 74*(2), 289–300. https://doi.org/10.1006/brln.2000.2313.

Friederici, A. D., Rüschemeyer, S.-A., Hahne, A., & Fiebach, C. J. (2003). The role of left inferior frontal and superior temporal cortex in sentence comprehension: Localizing syntactic and semantic processes. *Cerebral Cortex, 13*(2), 170–177. https://doi.org/10.1093/cercor/13.2.170.

Friston, K. J., Ashburner, J., Frith, C. D., Poline, J.-B., Heather, J. D., & Frackowiak, R. S. J. (1995). Spatial registrationand normalizationof images. *Human Brain Mapping, 2*, 165–189. https://doi.org/10.1002/hbm.460030303.

Friston, K. J., Holmes, A. P., Worsley, K. J., Poline, J.-P., Frith, C. D., & Frackowiak, R. S. J. (1995). Statistical parametric maps in functional imaging: A general linear approach. *Human Brain Mapping, 2*(4), 189–210. https://doi.org/10.1002/hbm.460020402.

Gibson, E. (1998). Linguistic complexity: Locality of syntactic dependencies. *Cognition, 68*(1), 1–76. https://doi.org/10.1016/S0010-0277(98)00034-1.

Gibson, E., & Wu, H. H. I. (2013). Processing Chinese relative clauses in context. *Language and Cognitive Processes, 28*(1–2), 1–31. https://doi.org/10.1080/01690965.2010.536656.

Goebela, R., Roebroecka, A., Kimb, D.-S., & Formisano, E. (2003). Investigating directed cortical interactions in time-resolved fMRI data using vector autoregressive modeling and Granger causality mapping. *Magnetic Resonance Imaging, 21*(10), 1251–1261. https://doi.org/10.1016/j.mri.2003.08.026.

Granger, C. W. J. (1969). Investigating causal relations by econometric models and cross-spectral methods. *Econometrica, 37*(3), 424–438. https://doi.org/10.2307/1912791.

Grodner, D., & Gibson, E. (2005). Consequences of the serial nature of linguistic input for sentenial complexity. *Cognitive Science, 29*(2), 261–290. https://doi.org/10.1207/s15516709cog0000_7.

Grodzinsky, Y., & Friederici, A. D. (2006). Neuroimaging of syntax and syntactic processing. *Current Opinion in Neurobiology, 16*(2), 240–246. https://doi.org/10.1016/j.conb.2006.03.007.

Hagoort, P., Indefrey, P., Brown, C., Herzog, H., Steinmetz, H., Seitz, R., et al. (1999). The neural circuitry involved in the reading of German words and pseudowords: A PET study. *Journal of Cognitive Neuroscience, 11*(4), 383–398. https://doi.org/10.1162/089892999563490.

Hagoort, P. (2014). Nodes and networks in the neural architecture for language: Broca's region and beyond. *Current Opinion in Neurobiology, 28*, 136–141. https://doi.org/10.1016/j.conb.2014.07.013.

Hartwigsen, G., Henseler, I., Stockert, A., Wawrzyniak, M., Wendt, C., Klingbeil, J., et al. (2017). Integration demands modulate effective connectivity in a fronto-temporal network for contextual sentence integration. *NeuroImage, 147*, 812–824. https://doi.org/10.1016/j.neuroimage.2016.08.026.

He, W., & Chen, B.-G. (2013). The role of animacy in Chinese relative clause processing. *Acta Psychol (Amst), 144*(1), 145–153. https://doi.org/10.1016/j.actpsy.2013.04.022.

Hsiao, F., & Gibson, E. (2003). Processing relative clauses in Chinese. *Cognition, 90*(1), 3–27. https://doi.org/10.1016/s0010-0277(03)00124-0.

Hsieh, Y., Boland, J. E., Zhang, Y., & Yan, M. (2009). Limited syntactic parallelism in Chinese ambiguity resolution. *Language and Cognitive Processes, 24*(7–8), 1227–1264. https://doi.org/10.1080/01690960802050375.

Hsu, N. C.-C., Hermon, G., & Zukowski, A. (2009). Young children's production of head-

final relative clauses: Elicited production data from Chinese children. *Journal of East Asian Linguistics, 18*(4), 23–60. https://doi.org/10.1007/s10831-009-9047-y.
Hutton, C., Bork, A., Josephs, O., Deichmann, R., Ashburner, J., & Turner, R. (2002). Image distortion correction in fMRI: A quantitative evaluation. *NeuroImage, 16*(1), 217–240. https://doi.org/10.1006/nimg.2001.1054.
Just, M. A., & Carpenter, P. A. (1992). A capacity theory of comprehension: Individual differences in working memory. *Psychological Review, 99*(1), 122.
Just, M. A., Carpenter, P. A., Keller, T. A., Eddy, W. F., & Thulborn, K. R. (1996). Brain activation modulated by sentence comprehension. *Science, 274*(5284), 114–116. https://doi.org/10.1126/science.274.5284.114.
Keller, T. A., Carpenter, P. A., & Just, M. A. (2001). The neural bases of sentence comprehension: A fMRI examination of syntactic and lexical processing. *Cerebral Cortex, 11*(3), 223–237. https://doi.org/10.1093/cercor/11.3.223.
Kemmerer, D. (2012). The cross-linguistic prevalence of SOV and SVO word orders reflects the sequential and hierarchical representation of action in Broca's area. *Language and Linguistics Compass, 6*(1), 50–66. https://doi.org/10.1002/lnc3.322.
Kim, J.-J., Kim, M. S., Lee, J. S., Lee, D. S., Lee, M. C., & Kwon, J. S. (2002). Dissociation of working memory processing associated with native and second languages: PET investigation. *NeuroImage, 15*(4), 879–891. https://doi.org/10.1006/nimg.2001.1025.
King, J., & Just, M. A. (1991). Individual differences in syntactic processing: The role of working memory. *Journal of Memory and Language, 30*(5), 580–602. https://doi.org/10.1016/0749-596X(91)90027-H.
King, J., & Kutas, M. (1995). Who did what and when? Using word- and clause-level ERPs to monitor working memory usage in reading. *Journal of Cognitive Neuroscience, 7*(3), 376–395. https://doi.org/10.1162/jocn.1995.7.3.376.
Kuhnke, P., Meyer, L., Friederici, A. D., & Hartwigsen, G. (2017). Left posterior inferior frontal gyrus is causally involved in reordering during sentence processing. *NeuroImage, 148*, 254–263. https://doi.org/10.1016/j.neuroimage.2017.01.013.
Leff, A. P., Schofield, T. M., Crinion, J. T., Seghier, M. L., Grogan, A., Green, D. W., et al. (2009). The left superior temporal gyrus is a shared substrate for auditory short-term memory and speech comprehension: Evidence from 210 patients with stroke. *Brain, 132*(12), 3401–3410. https://doi.org/10.1093/brain/awp273.
Lin, C.-J. C. (2014). Effect of thematic order on the comprehension of Chinese relative clauses. *Lingua, 140*, 180–206. https://doi.org/10.1016/j.lingua.2013.12.003.
Lin, Y., & Garnsey, S. (2011). Animacy and the resolution of temporary ambiguity in relative clause comprehension in Mandarin. In H. Yamashita, Y. Hirose, & J. L. Packard (Eds.). *Processing and producing head-final structures* (pp. 241–275). New York: Springer.
Mason, R. A., Just, M. A., Keller, T. A., & Carpenter, P. A. (2003). Ambiguity in the brain: What brain imaging reveals about the processing of syntactically ambiguous sentences. *Journal of Experimental Psychology, 29*(6), 1319–1338. https://doi.org/10.1037/0278-7393.29.6.1319.
Meyer, L., Obleser, J., Anwander, A., & Friederici, A. D. (2012). Linking ordering in Broca's area to storage in left temporo-parietal regions: The case of sentence processing. *NeuroImage, 62*(3), 1987–1998. https://doi.org/10.1016/j.neuroimage.2012.05.052.
Meyer, L., Grigutsch, M., Schmuck, N., Gaston, P., & Friederici, A. D. (2015). Frontal-posterior theta oscillations reflect memory retrieval during sentence comprehension. *Cortex, 71*, 205–218. https://doi.org/10.1016/j.cortex.2015.06.027.
Musso, M., Moro, A., Glauche, V., Rijntjes, M., Reichenbach, J., Büchel, C., et al. (2003). Broca's area and the language instinct. *Nature Neuroscience, 6*(7), 774–781. https://doi.org/10.1038/nn1077.
Nakano, Y., Felser, C., & Clahsen, H. (2002). Antecedent priming at trace positions in Japanese long-distance scrambling. *Journal of Psycholinguistic Research, 31*, 531–571.
Newman, A. J., Pancheva, R., Ozawa, K., Neville, H. J., & Ullman, M. T. (2001). An event-related fMRI study of syntactic and semantic violations. *Journal of Psycholinguistic Research, 30*(3), 339–364. https://doi.org/10.1023/A:1010499119393.
Nishitani, N., Schürmann, M., Amunts, K., & Hari, R. (2005). Broca's region: From action to language. *Physiology, 20*(1), 60–69. https://doi.org/10.1152/physiol.00043.2004.
Novais-Santos, S., Gee, J., Shah, M., Troiani, V., Work, M., & Grossman, M. (2007). Resolving sentence ambiguity with planning and working memory resources: Evidence from fMRI. *NeuroImage, 37*(1), 361–378. https://doi.org/10.1016/j.neuroimage.2007.03.077.
Opitz, B., & Friederici, A. D. (2004). Brain correlates of language learning: The neuronal dissociation of rule-based versus similarity-based learning. *Journal of Neuroscience, 24*(39), 8436–8440. https://doi.org/10.1523/JNEUROSCI.2220-04.2004.
Owen, A. M., McMillan, K. M., Laird, A. R., & Bullmore, E. (2005). N-back working memory paradigm: A meta-analysis of normative functional neuroimaging studies. *Human Brain Mapping, 25*(1), 46–59. https://doi.org/10.1002/hbm.20131.
Packard, J. L., Ye, Z., & Zhou, X. (2010). Filler-Gap Processing in Mandarin Relative Clauses: Evidence from Event-Related Potentials. In H. Yamashita, Y. Hirose, & J. L. Packard (Eds.). *Processing and Producing Head-final Structures* (pp. 219–240). New York: Springer.
Papoutsi, M., Stamatakis, E. A., Griffiths, J., Marslen-Wilson, W. D., & Tyler, L. K. (2011). Is left fronto-temporal connectivity essential for syntax? Effective connectivity, tractography and performance in left-hemisphere damaged patients. *NeuroImage, 58*, 656–664. https://doi.org/10.1016/j.neuroimage.2011.06.036.
Pattamadilok, C., Dehaene, S., & Pallier, C. (2016). A role for left inferior frontal and posterior superior temporal cortex in extracting a syntactic tree from a sentence. *Cortex, 75*, 44–55. https://doi.org/10.1016/j.cortex.2015.11.012.
Phillips, C., Kazanina, N., & Abada, S. H. (2005). ERP effects of the processing of syntactic long-distance dependencies. *Cognitive Brain Research, 22*(3), 407–428. https://doi.org/10.1016/j.cogbrainres.2004.09.012.
Pu, M. M. (2007). The distribution of relative clauses in Chinese discourse. *Discourse Processes, 43*(1), 25–53. https://doi.org/10.1207/s15326950dp4301_2.
Pugh, K. R., Shaywitz, B. A., Shaywitz, S. E., Shankweiler, D. P., Katz, L., Fletcher, J. M., et al. (1996). Predicting reading performance from neuroimaging profiles: The cerebral basis of phonological effects in printed word identification. *NeuroImage, e(e)*, S455. https://doi.org/10.1016/S1053-8119(96)80457-5.
Qiao, X., Shen, L., & Forster, K. (2012). Relative clause processing in Mandarin: Evidence from the maze task. *Language and Cognitive Processes, 27*(4), 611–630. https://doi.org/10.1080/01690965.2011.578394.
Roebroeck, A., Formisano, E., & Goebel, R. (2005). Mapping directed influence over the brain using Granger causality and fMRI. *NeuroImage, 25*(1), 230–242. https://doi.org/10.1016/j.neuroimage.2004.11.017.
Rodd, J. M., Longe, O. A., Randall, B., & Tyler, L. K. (2010). The functional organisation of the fronto-temporal language system: Evidence from syntactic and semantic ambiguity. *Neuropsychologia, 48*(5), 1324–1335. https://doi.org/10.1016/j.neuropsychologia.2009.12.035.
Rogalsky, C., & Hickok, G. (2010). The role of Broca's area in sentence comprehension. *Journal of Cognitive Neuroscience, 23*(7), 1664–1680. https://doi.org/10.1162/jocn.2010.21530.
Rogalsky, C., Matchin, W., & Hickok, G. (2008). Braoca's area, sentence comprehension, and working memory: An fMRI study. *Frontiers in Human Neuroscience, 2*, 1–13. https://doi.org/10.3389/neuro.09.014.2008.
Sakai, K. L. (2005). Language acquisition and brain development. *Science, 310*(5749), 815–819. https://doi.org/10.1126/science.1113530.
Santi, A., & Grodzinsky, Y. (2010). fMRI adaptation dissociates syntactic complexity dimensions. *NeuroImage, 51*, 1285–1293. https://doi.org/10.1016/j.neuroimage.2010.03.034.
Sato, J. R., Takahashi, D. Y., Arcuri, S. M., Sameshima, K., Morettin, P. A., & Baccalá, L. A. (2009). Frequency domain connectivity identification: An application of partial directed coherence in fMRI. *Human Brain Mapping, 30*(2), 452–461. https://doi.org/10.1002/hbm.20513.
Skeide, M. A., Brauer, J., & Friederici, A. D. (2014). Syntax gradually segregates from semantics in the developing brain. *NeuroImage, 100*, 106–111. https://doi.org/10.1016/j.neuroimage.2014.05.080.
Skeide, M. A., Brauer, J., & Friederici, A. D. (2016). Brain Functional and Structural Predictors of Language Performance. *Cerebral Cortex, 26*, 2127–2139. https://doi.org/10.1093/cercor/bhv042.
Smith, E. E., & Jonides, J. (1998). Neuroimaging analyses of human working memory. *Proceedings of the National Academy of Sciences, 85*(20), 12061–12068. https://doi.org/10.1073/pnas.95.20.12061.
Sreenivasan, K. K., Curtis, C. E., & D' Esposito, M. (2014). Revisiting the role of persistent neural activity during working memory. *Trends in Cognitive Science, 18*(2), 82–89. https://doi.org/10.1016/j.tics.2013.12.001.
Stowe, L. A., Broere, C. C. A. J., Paans, A. M. J., Wijers, A. A., Mulder, G., Vaalburg, W., et al. (1998). Localizing components of a complex task: Sentence processing and working memory. *Neuroreport, 9*(13), 2995–2999. https://doi.org/10.1097/00001756-199809140-00014.
Stromswold, K., Caplan, D., Alpert, N., & Rauch, S. (1996). Localization of syntactic comprehension by positron emission tomography. *Brain and Language, 52*(3), 452–473. https://doi.org/10.1006/brln.1996.0024.
Sung, Y.-T., Cha, J.-H., Tu, J.-Y., Wu, M.-D., & Lin, W.-C. (2016). Investigating the Processing of Relative Clauses in Mandarin Chinese: Evidence from Eye-Movement Data. *Journal of Psycholinguistic Research, 45*(5), 1089–1113. https://doi.org/10.1007/s10936-015-9394-y.
Tettamanti, M., Alkadhi, H., Moro, A., Perani, D., Kollias, S., & Weniger, D. (2002). Neural correlates for the acquisition of natural language syntax. *NeuroImage, 17*(2), 700–709. https://doi.org/10.1006/nimg.2002.1201.
Traxler, M. J., Morris, R. K., & Seely, R. E. (2002). Processing subject and object relative clauses: Evidence from eye movements. *Journal of Memory and Language, 47*(1), 69–90. https://doi.org/10.1006/jmla.2001.2836.
Ueno, M., & Kluender, R. (2003). Event-related brain indices of Japanese scrambling. *Brain and Language, 86*(2), 243–271. https://doi.org/10.1016/S0093-934X(02)00543-6.
Vasishth, S., Chen, Z., Li, Q., & Guo, G. (2013). Processing Chinese relative clauses: Evidence for the subject-relative advantage. *PLoS One, 8*(10), e77006. https://doi.org/10.1371/journal.pone.0077006.
Waters, G. S., & Caplan, D. (1996). The measurement of verbal working memory capacity and its relation to reading comprehension. *The Quarterly Journal of Experimental Psychology Section A, 49*(1), 51–79. https://doi.org/10.1080/713755607.
Wu, F., Kaiser, E., & Andersen, E. (2012). Animacy effects in Chinese relative clause processing. *Language and Cognitive Processes, 27*(10), 1489–1524. https://doi.org/10.1080/01690965.2011.614423.
Xu, K., Duann, J. R., Hung, D. L., & Wu, D. H. (2019). Preference for Object Relative Clauses in Chinese Sentence Comprehension: Evidence from Online Self-paced Reading Time. *Frontiers in Psychology, 10*, 2210. https://doi.org/10.3389/fpsyg.2019.02210.
Yang, C. L., & Perfetti, C. A. (2006). Contextual constraints on the comprehension of relative clause sentences in Chinese: ERPs evidence. *Language and Linguistics, 7*(3), 697–730.
Yang, C. L., Perfetti, C. A., & Liu, Y. (2010). Sentence integration processes: An ERP study of Chinese sentence comprehension with relative clauses. *Brain and Language, 112*, 85–100. https://doi.org/10.1016/j.bandl.2009.10.005.
Zempeni, M. Z., Renken, R., Hoeks, J. C. J., Hoogduin, J. M., & Stowe, L. A. (2007). Semantic ambiguity processing in sentence context: Evidence from event-related fMRI. *NeuroImage, 34*(3), 1270–1279. https://doi.org/10.1016/j.neuroimage.2006.09.048.
Zhang, Y. X., Zhang, H. C., & Hua, S. (2000). A study on the processing of ambiguous phrases in Chinese. *Chinese Journal of Psychology, 32*, 13–19. https://doi.org/10.3724/SP.J.1041.2018.01323.
